# Neurovascular Uncoupling in Alzheimer’s and Parkinson’s Diseases: Mechanisms and Therapeutic Strategies

**DOI:** 10.3390/brainsci16050434

**Published:** 2026-04-22

**Authors:** Akash Ved, Tushar B. Gajjar, Ashish Kakkad, Subbulakshmi Ganesan, Aman Shankhyan, Karthikeyan Jayabalan, Swati Mishra, Bhavik Jain, Vimal Arora, Monica Gulati, Tapan Behl, Ansab Akhtar

**Affiliations:** 1 Faculty of Pharmacy, Dr. APJ Abdul Kalam Technical University, Lucknow 226031, Uttar Pradesh, India; akashved@gmail.com; 2 Faculty of Pharmacy, Gokul Global University, Sidhpur 384151, Gujarat, India; tbgajjar.gphc@gokuluniversity.ac.in; 3 Marwadi University Research Center, Department of Physiotherapy, Faculty of Health Sciences, Marwadi University, Rajkot 360003, Gujarat, India; ashish.kakkad@marwadieducation.edu.in; 4 Department of Chemistry and Biochemistry, School of Sciences, JAIN (Deemed to be University), Bangalore 560041, Karnataka, India; g.subbulakshmi@jainuniversity.ac.in; 5 Centre for Research Impact & Outcome, Institute of Engineering and Technology, Chitkara University, Rajpura 140401, Punjab, India; aman_shankhyan@outlook.com; 6 Department of Chemistry, Sathyabama Institute of Science and Technology, Chennai 600119, Tamil Nadu, India; karthikeyan.chemistry@sathyabhama.ac.in; 7 Department of Pharmacology, IMS and SUM Hospital, Siksha ‘O’ Anusandhan (Deemed to be University), Bhubaneswar 751003, Odisha, India; swati.sihara@soa.ac.in; 8 Sharda School of Pharmacy, Sharda University, Greater Noida 201310, Uttar Pradesh, India; bhavik192024@outlook.com; 9 University Institute of Pharmacy Sciences, Chandigarh University, Mohali 140413, Punjab, India; drvimalarora1@outlook.com; 10 School of Pharmaceutical Sciences, Lovely Professional University, Phagwara 144411, Punjab, India; monicagulati14@gmail.com; 11 Australian Research Centre in Complementary and Integrative Medicine (ARCCIM), Faculty of Health, University of Technology Sydney, Ultimo, NSW 2007, Australia; 12 Amity Institute of Pharmacy, Amity University Punjab, Mohali 140306, Punjab, India; 13 LSU Health Sciences Center, School of Medicine, LSU Health New Orleans, New Orleans, LA 70112, USA

**Keywords:** neurovascular uncoupling, Alzheimer’s disease, Parkinson’s disease, blood–brain barrier dysfunction, cerebral blood flow regulation

## Abstract

**Highlights:**

**What are the main findings?**

• Neurovascular dysfunction contributes to AD & PD through BBB disruption and neuroinflammation.
• Multi-omics and neuroimaging technologies enable early detection and monitoring of neurovascular dysfunction.

**What are the implications of the main findings?**

• Interventions targeting endothelial dysfunction and neuroinflammation show potential for treating AD and PD.
• Cross-disciplinary collaboration is key to advancing neurovascular uncoupling research and developing novel therapies.

**Abstract:**

Neurovascular coupling (NVC) maintains appropriate cerebral blood flow (CBF) in response to neuronal activity, and its disturbance, known as neurovascular uncoupling (NVU), is increasingly recognised as a major contributor to neurodegenerative disease. Alzheimer’s disease (AD) NVU is caused by Aβ buildup, tau pathology, endothelial dysfunction, and persistent neuroinflammation, leading to poor CBF control and blood–brain barrier (BBB) disintegration. Parkinson’s disease (PD) is characterised by α-synuclein aggregation, oxidative stress, mitochondrial dysfunction, and dopaminergic neuronal loss, all of which impede cerebrovascular regulation. These disease-specific mechanisms interact via similar vascular pathways, establishing NVU as a critical connection between neuronal degeneration and cerebrovascular dysfunction. This study highlights the critical role of NVU in neurodegeneration by investigating shared and disease-specific processes in AD and PD. Tau pathology disturbs vascular regulation in AD, whereas dopaminergic neuron loss impairs cerebrovascular control in PD. Both Aβ and α-synuclein are linked to endothelial dysfunction and oxidative stress, albeit originating in different pathologies. Comparative analysis reveals distinct vascular abnormalities in each condition, as well as shared processes such as inflammation and BBB disruption. The study also covers developments in biomarker discovery and neuroimaging techniques that allow for exact characterisation of NVU, facilitating early diagnosis and treatments. In addition, lifestyle changes and pharmacological treatments for oxidative stress and endothelial injury are being examined. This study highlights the significance of NVU as a fundamental pathogenic mechanism, underscoring its importance for comprehending disease development and formulating novel therapeutic strategies.

## 1. Introduction

Neurovascular coupling (NVC) is the regulatory process that matches cerebral blood flow (CBF) to neuronal metabolic demand. By dynamically adjusting vascular responses to neural activity, NVC ensures adequate delivery of oxygen and nutrients, thereby maintaining neuronal function and cerebral homeostasis [1]. The neurovascular unit (NV Unit) is a highly specialized cellular network that includes neurons, astrocytes, pericytes, endothelial cells, and vascular smooth muscle cells. It is essential for the coordination of this response [2]. The NV unit regulates CBF via vessel dilation and constriction through NVC; its disruption, neurovascular uncoupling (NVU), significantly contributes to Alzheimer’s disease (AD) and Parkinson’s disease (PD) [3,4,5]. Insufficient blood supply and metabolic support result from NVC’s inability to maintain neuronal-vascular balance, accelerating neurodegeneration and increasing neuronal vulnerability [6,7].

Amyloid-β (Aβ) deposition and tau aggregation drive NVU in AD, impairing NV unit integrity, inducing endothelial dysfunction, reducing vasodilation, and disrupting blood–brain barrier (BBB) function [8,9]. At the same time, tau pathology exacerbates damage to the NV unit by encouraging oxidative stress and neuroinflammation, which leads to vascular dysregulation. A key element of the NV unit, the BBB, breaks down, making it easier for neurotoxic chemicals to seep into the brain and setting off a chain reaction of inflammation and neuronal damage. These vascular alterations play a crucial part in the early stages of AD development because they occur before the emergence of clinical symptoms. In PD, NVU is caused by distinct but overlapping pathways [10,11]. The aggregation of α-synuclein, a pathological hallmark of PD, has been associated with cerebrovascular dysfunction. α-synuclein inhibits endothelial cell function and affects pericyte-endothelial cell signalling, which is crucial for maintaining the BBB and controlling CBF [12]. Furthermore, the increasing loss of dopaminergic neurons in PD interferes with the precise brain control of vascular responses [13]. Mitochondrial failure, a well-documented hallmark of PD, exacerbates oxidative stress and energy deficiencies in NV unit components, further impairing vascular function. These processes are exacerbated by persistent neuroinflammation, which affects both the neuronal and vascular components of the NV unit [14,15].

Despite its apparent role in both AD and PD, NVU remains an underexplored therapeutic target. Traditional approaches to neurodegeneration have mostly focused on neuronal pathology, sometimes disregarding the important role of vascular dysfunction. However, mounting data suggest that NVU is not just a spectator but an active cause of illness progression. The reciprocal link between neuronal and vascular dysfunction results in a vicious cycle in which defective vascular control worsens neuronal damage and vice versa [3,16]. Targeting NVU may disrupt pathological cycles, benefiting neuronal and vascular health. Across AD and PD, the major pathogenic pathways unfold through a common sequence of protein modification, oxidative stress, endothelial/pericyte injury, BBB breakdown, and impaired nitric oxide (NO)-dependent vascular signaling, ultimately producing NVU. This review examines NVU mechanisms in AD and PD, highlighting shared pathways (oxidative stress, inflammation, BBB disruption) and disease-specific drivers (amyloid-β, tau, α-synuclein, dopaminergic loss) underlying vascular dysfunction [2,3].

## 2. Pathophysiology of NVU

NVU results from coordinated molecular and cellular disruptions, principally due to oxidative stress, neuroinflammation, and poor vasoactive signalling. Excessive generation of reactive oxygen species (ROS) harms lipids, proteins, and DNA, with endothelial cells and pericytes especially sensitive. This injury weakens the BBB, allowing neurotoxins to enter the brain and exacerbating neuronal degeneration due to persistent vascular dysfunction [17,18].

Among the various mechanisms contributing to NVU, dysregulation of NO signaling plays a central integrative role linking neuronal activity to vascular responses. Endothelial dysfunction drives NVU by reducing NO bioavailability, impairing vasodilation, limiting oxygen delivery, promoting inflammatory cytokines, adhesion molecules, immune infiltration, and compromising NV unit integrity [19,20,21]. NO signaling operates as a key integrative mediator of NVC by coordinating communication across neurons, endothelial cells, astrocytes, and pericytes. Neurons generate NO via neuronal nitric oxide synthase (nNOS) in response to synaptic activity, which diffuses to adjacent endothelial cells and vascular smooth muscle cells to induce vasodilation. Endothelial cells further amplify this response through endothelial nitric oxide synthase (eNOS), while astrocytes modulate NO signaling indirectly via calcium-dependent release of vasoactive mediators such as prostaglandins and arachidonic acid metabolites. Pericytes also respond to NO gradients, regulating capillary diameter and local blood flow. Importantly, this coordinated NO signaling represents a dynamic intercellular network rather than an isolated endothelial mechanism, integrating neuronal activity with vascular responses across the NV unit. Experimental approaches such as single-cell transcriptomics, spatial omics, and NVU co-culture or organ-on-chip models may further clarify cell-type-specific NO signaling dynamics and their contribution to NVU. Disruption of this coordinated NO signaling network impairs intercellular communication within the NV unit, leading to defective NVC and reduced cerebral perfusion [22,23]. Single-cell transcriptomics and spatial omics can identify cell-type-specific nitric oxide synthase (NOS) expression patterns, while optogenetic and chemogenetic tools may enable selective modulation of nNOS, eNOS, and iNOS activity in neurons, endothelial cells, and glia. Additionally, organ-on-chip and co-culture NVU models provide controlled platforms to study dynamic NO signaling between astrocytes, pericytes, and endothelial cells. These approaches will help clarify how NO-dependent signaling integrates neuronal activity with vascular responses under physiological and pathological conditions [24]. Importantly, this NO-dependent signaling network does not function in isolation but is tightly coordinated with astrocyte-mediated neurovascular communication. By converting synaptic information into CBF regulation, astrocytes play a crucial role as mediators between neuronal activity and cerebrovascular responses. The buildup of neurotoxic substances like α-synuclein or Aβ interferes with astrocytic calcium signalling during NVC, making it harder for them to coordinate vascular responses and ultimately leading to impaired neurovascular communication [25,26]. Astrocyte dysfunction promotes pro-inflammatory mediators, sustaining NV units inflammation. Pericytes, essential for BBB integrity, are vulnerable to oxidative stress; their loss disrupts endothelial-pericyte signalling, increasing permeability, impairing CBF, and extracellular matrix remodelling [26,27,28]. NVU directly contributes to neurodegeneration through a bidirectional relationship between vascular and neuronal dysfunction. Insufficient CBF resulting from NVU deprives neurons of the oxygen and nutrients required to sustain synaptic activity and cellular metabolism. This metabolic stress renders neurons vulnerable to excitotoxicity, mitochondrial dysfunction, and apoptosis [29,30]. By interfering with neurovascular communication, neuronal impairment intensifies vascular dysfunction and creates a vicious cycle of degradation. At the same time, prolonged microglial activation increases NVU by releasing pro-inflammatory cytokines and ROS, which accelerate neuronal injury and harm components of the NV unit [31,32]. Microglial dysfunction, combined with a compromised BBB, facilitates the infiltration of peripheral immune cells into the brain, amplifying inflammation and tissue damage. The relationship between NVU and neurodegeneration is also mediated by alterations in key signalling pathways. For example, vascular endothelial growth factor (VEGF), which is essential for angiogenesis and BBB maintenance, becomes dysregulated in NVU [33,34]. Overexpression of VEGF can lead to pathological angiogenesis and BBB leakage, while insufficient VEGF activity results in endothelial cell death and impaired vascular repair. Additionally, signalling pathways involving transforming growth factor-beta (TGF-β) and matrix metalloproteinases (MMPs) are disrupted in NVU, contributing to ECM degradation and the NVC destabilization [35]. Elucidating these interrelated pathways is critical for developing targeted therapies to restore neurovascular integrity and delay neurological deterioration [36,37]. Overall, these interconnected mechanisms demonstrate how coordinated dysfunction across NV unit components drives NVU and promotes neurodegenerative progression.

## 3. NVU in AD

**Figure 1:**
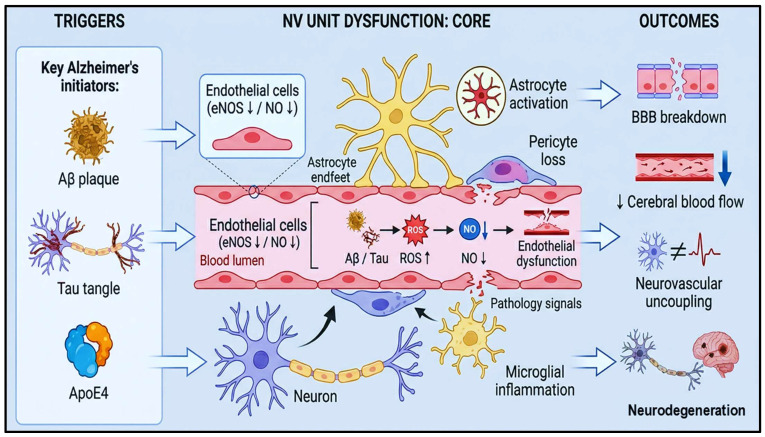
Neurovascular Uncoupling in Alzheimer’s Disease. This Figure illustrates how Aβ plaques, tau tangles, and ApoE4 trigger endothelial cell dysfunction, reduced eNOS activity, and NO loss within the BBB. Aβ/tau-induced ROS promote endothelial injury, astrocyte activation, pericyte loss, and microglial inflammation. These changes disrupt astrocyte endfeet support, impair cerebral blood flow, and culminate in NVU and neurodegeneration. Abbreviations: Aβ: amyloid-β; ApoE4: apolipoprotein E4; BBB: blood–brain barrier; eNOS: endothelial nitric oxide synthase; NO: nitric oxide; ROS: reactive oxygen species; NVU: neurovascular unit.

AD-related neurovascular dysfunction is driven by Aβ, tau, endothelial injury, BBB disruption, and inflammation [37,38] (Figure 1).

### 3.1. Role of Amyloid-β in NVU

Aβ is a primary pathological hallmark of AD and a critical contributor to NVU. Aβ exerts its effects on the NV unit through multiple mechanisms that compromise its cellular and molecular components, ultimately impairing CBF regulation and BBB integrity. One of the most detrimental effects of Aβ is its interaction with endothelial cells, the key regulators of vascular tone and BBB function [39,40]. Perivascular Aβ accumulation increases ROS in endothelial cells, causing oxidative injury and reduced NO, impairing vasodilation, disrupting NVC, and brain perfusion [39,41]

Furthermore, Aβ disrupts pericytes, specialized mural cells that play a pivotal role in maintaining BBB integrity and regulating capillary diameter. Aβ interacts with low-density lipoprotein receptor-related protein 1 (LRP1) receptors on pericytes, triggering their apoptosis and detachment from the endothelial layer. Pericyte loss results in capillary constriction, further reducing CBF, and destabilizing the BBB. The weakened BBB permits the infiltration of circulating Aβ and other neurotoxic molecules, exacerbating neurovascular damage. Astrocytes, another critical component of the NV units, are also adversely affected by Aβ [42,43]. Normally, astrocytes regulate vascular responses via vasoactive molecules (arachidonic acid metabolites). Aβ disrupts astrocytic Ca^2+^ signalling, impairs CBF regulation, induces reactive astrocytosis, releases cytokines/chemokines, exacerbating neuroinflammation and damaging NV Unit integrity [26,44,45]. Aβ negatively affects smooth muscle cells in the brain, which are responsible for regulating blood flow and vascular tone. The degeneration of these cells promotes arterial stiffness and disturbs cerebral autoregulation, limiting the ability of cerebral arteries to alter perfusion in response to changing metabolic needs [46,47].

Aβ triggers inflammation by activating pattern recognition receptors, such as toll-like receptors and RAGE, on microglia and endothelial cells. This relationship increases prolonged cytokine and adhesion molecule release, exacerbating vascular inflammation. The subsequent cascade increases oxidative stress, compromises endothelial tight junction integrity, and promotes peripheral immune cell infiltration, exacerbating NVU [48,49]. Soluble Aβ oligomers impair NV Unit by disrupting synaptic signaling, mitochondrial function, and neuron-vascular communication, causing energy deficits, reduced CBF regulation, and compromised BBB integrity, beyond extracellular Aβ aggregates [16,50].

Collectively, Aβ pathology creates a self-perpetuating cycle of neurovascular dysfunction in AD. The initial vascular deficits caused by Aβ deposition exacerbate hypoxia and metabolic stress in the brain, which, in turn, promote further Aβ production and aggregation. This vicious cycle underscores the central role of Aβ in driving NVU and highlights its significance as a therapeutic target. Strategies aimed at reducing Aβ toxicity, restoring endothelial function, and preserving pericyte viability hold promise for mitigating the vascular contributions to AD progression [51,52].

### 3.2. Impact of Tau Pathology on Cerebrovascular Function

Beyond Aβ pathology, tau-mediated mechanisms further exacerbate neurovascular dysfunction. Tau pathology significantly contributes to NVU in AD and tauopathies, where specific hyperphosphorylated tau species, including AT8 (Ser202/Thr205), PHF-1 (Ser396/Ser404), and Thr231-phosphorylated tau, disrupt multiple NV unit components [53,54], causing BBB impairment and reduced CBF [55,56]. Among these, AT8-positive tau is particularly associated with early neurovascular dysfunction and correlates with impaired CBF and endothelial abnormalities, while PHF-1-associated tau species are linked to advanced neurofibrillary pathology and vascular instability [53,54]. Tau pathology disrupts axonal transport via microtubule dysfunction, impairing delivery of signaling molecules, leading to defective NVC, reduced CBF, hypoperfusion, and metabolic oxygen mismatch in neurodegeneration [57,58,59].

Tau affects astrocytes, disrupting calcium signalling, impairing NV unit integrity, and CBF regulation. This reduces vasodilation, alters prostaglandins and arachidonic acid release, and impairs neurovascular coordination and cerebrovascular responsiveness [57,60,61]. Astrocytic activation releases TNF-α and IL-1β, driving neuroinflammation and endothelial dysfunction. Tau accumulation increases ROS, activates inflammatory pathways, reduces NO bioavailability, and impairs endothelial-dependent vasodilation, promoting vascular dysfunction [26,27,62]. Mechanistically, hyperphosphorylated tau may impair the NO system by increasing ROS production, promoting eNOS uncoupling, and enhancing NO scavenging by superoxide, thereby reducing NO bioavailability and favoring peroxynitrite formation. Phosphorylation at Thr231 and Ser396/404 has been specifically implicated in disrupting microtubule stability and endothelial signaling, which indirectly affects NO-mediated vasodilation and vascular responsiveness [63]. In parallel, tau-associated neuroinflammation may upregulate iNOS, contributing to dysregulated NO signaling and endothelial injury. Loss of NO bioavailability impairs vascular reactivity by reducing endothelial-dependent vasodilation. NO normally activates soluble guanylate cyclase (sGC) in vascular smooth muscle cells, increasing cyclic guanosine monophosphate (cGMP) levels and promoting relaxation. Reduced NO levels, often due to oxidative stress-mediated scavenging by superoxide radicals, limit this signalling pathway, leading to impaired vasodilation, increased vascular stiffness, and reduced CBF [64,65]. Tau accumulation in pericytes impairs contractility, causing detachment, capillary constriction, reduced CBF, increased BBB permeability, hypoperfusion, and vascular instability in AD, while disrupting basement membrane and extracellular matrix integrity [66,67,68,69]. Given this mechanistic link between tau pathology and impaired NO signaling, targeting the NO-tau axis represents a rational therapeutic strategy. In this context, NO-targeted interventions have emerged as promising approaches to modulate tau pathology and restore neurovascular function. NO-related interventions, such as S-nitrosoglutathione (GSNO), have been shown to attenuate tau hyperphosphorylation by inhibiting GSK-3β and cyclin-dependent kinase 5 (CDK5) activity. Additionally, modulation of NOS activity and scavenging of reactive nitrogen species may prevent peroxynitrite-mediated tau nitration and aggregation. Pharmacological inhibitors of GSK-3β (e.g., lithium) and CDK5, along with antioxidant compounds such as resveratrol, further support the therapeutic potential of targeting NO-mediated tau pathology in neurodegenerative diseases [70,71,72]. Thus, modulation of NO-dependent pathways may simultaneously attenuate tau pathology and restore vascular signaling, highlighting the NO-tau axis as a key therapeutic target in neurodegenerative disease.

Tau activates microglia via TLRs and RAGE, inducing IL-6 and TNF-α release, impairing endothelial function, disrupting the BBB, increasing oxidative stress, recruiting immune cells, and exacerbating NVU [73,74,75].

Tau disease gradually damages many NV Unit components, including endothelial cells, pericytes, astrocytes, and microglia, resulting in a cumulative impairment of neurovascular function in tauopathy. This degradation impairs CBF regulation and initiates a self-sustaining loop of oxidative stress and neuroinflammation, hastening neurodegenerative progression. As a result, understanding AD and associated conditions requires a better understanding of tau-driven vascular pathology. Therapeutic techniques focused on restoring endothelial integrity, pericyte stability, and astrocytic signalling may thus provide potential pathways for preventing cognitive decline and slowing disease development [76,77,78].

Similar to tau, phosphorylation-dependent modifications of other pathogenic proteins also contribute to neurovascular dysfunction. In PD, phosphorylation of α-synuclein at Ser129 is a critical pathological event that promotes aggregation, endothelial dysfunction, and BBB disruption. Phosphorylated α-synuclein has been shown to impair endothelial signaling, increase oxidative stress, and alter pericyte-endothelial interactions, thereby contributing to NVU. Additionally, dysregulated phosphorylation of proteins involved in endothelial signaling pathways, including eNOS and components of tight junction complexes, may further exacerbate vascular dysfunction in neurodegenerative diseases [79,80].

### 3.3. Endothelial Dysfunction and BBB Disruption in AD

Progressive cognitive impairment in AD is strongly linked to endothelial dysfunction and reduced BBB integrity. In AD, endothelial dysfunction leads to disruption of BBB integrity, resulting in increased permeability and impaired molecular exchange. Disruption of this vascular interface is a key pathogenic event that contributes to neurodegenerative development [81,82]. In AD, endothelial dysfunction and BBB integrity are impaired due to structural degeneration of endothelial cells, increasing BBB permeability, allowing fibrinogen and albumin leakage into the brain parenchyma, contributing to neurotoxicity and neuronal damage [82,83,84]. In AD, disruption of essential endothelial tight junction proteins such as ZO-1, claudin-5, and occludin affects BBB integrity, resulting in increased permeability and decreased control of nutrient transport into the brain. Concurrently, chronic neuroinflammatory signalling exacerbates endothelial dysfunction, strengthening vascular instability and hastening neurodegenerative development [26,85]. In AD, activated endothelial cells express adhesion molecules enabling leukocyte infiltration, exacerbating neuroinflammation and NV unit injury. Inflammatory mediators disrupt neuronal function, while reduced CBF impairs glucose and oxygen delivery, contributing to neuronal injury and cognitive deficits [86,87]. In addition to structural disruption of the BBB, alterations in nutrient transport mechanisms critically contribute to neurovascular dysfunction. Reduced expression of glucose transporter 1 (GLUT1) at the BBB impairs glucose transport into the brain, limiting substrate availability for glycolysis and oxidative phosphorylation. This results in decreased ATP production, mitochondrial dysfunction, and impaired synaptic activity, ultimately leading to cerebral hypometabolism and metabolic imbalance in AD [88,89,90,91,91]. Therefore, GLUT1 deficiency provides a direct mechanistic link between vascular dysfunction and neuronal energy failure in AD. Additionally, Aβ triggers inflammatory pathways that exacerbate endothelial dysfunction. The role of apolipoprotein E (ApoE), particularly the ε4 allele associated with increased risk for AD, is also critical. ApoE4 influences endothelial cell function by promoting inflammatory responses and reducing Aβ clearance across the BBB. In contrast, other isoforms such as ApoE2, may help maintain BBB integrity by inhibiting inflammation [92,93]. NVU contributes to BBB disruption in AD, where endothelial cells, pericytes, astrocytes, and neurons exhibit altered interactions, promoting neuroinflammation, neuronal death, and progressive cognitive decline through a vicious cycle [40,93]. Understanding these relationships has significant clinical implications for AD management. Targeting endothelial dysfunction and restoring BBB integrity could provide new therapeutic strategies aimed at slowing disease progression or improving cognitive outcomes. Potential approaches may include modulating inflammatory pathways, enhancing Aβ clearance mechanisms, or repairing damaged endothelial structures [94,95].

### 3.4. Neuroinflammation and Oxidative Stress in NVU

Oxidative stress represents another critical mechanism linking vascular dysfunction to neurodegeneration. ROS exert concentration-dependent effects on cellular signaling pathways. At low to moderate levels, ROS act as signaling molecules that activate pro-survival pathways such as PI3K/Akt and ERK1/2, supporting cellular adaptation and vascular homeostasis. In contrast, excessive ROS levels trigger pathological signaling cascades, including nuclear factor kappa B (NF-κB), c-Jun N-terminal kinase (JNK), and p38 MAPK pathways, leading to inflammation, apoptosis, and neurovascular dysfunction [96]. This dual role highlights the fine balance between physiological redox signaling and pathological oxidative stress within the NV unit. Under sustained pathological conditions, this balance is disrupted, and ROS activate the Nox4-dependent inflammatory cascade, promote MMP-9-mediated BBB disruption, and reduce NO bioavailability, thereby impairing vascular signaling [97]. Nox4 increases during hypoxia, and in pericytes induces MMP-9, promoting BBB breakdown and ischaemic injury [98,99]. Neuroinflammation, driven by oxidative stress, disrupts the NV unit via microglia and astrocyte-derived mediators, compromising the BBB, promoting neutrophil adhesion, amplifying inflammation, and forming a self-sustaining loop leading to NVU [37,100]. Neuroinflammation and oxidative stress are mutually reinforcing, with inflammatory signalling increasing ROS generation, which in turn sustains more inflammation and cellular damage. This pathogenic synergy in AD involves NVU-mediated BBB breakdown, permitting neurotoxin entry, neuronal injury, and cognitive impairment, while endothelial tight junction loss increases permeability, enabling inflammatory mediators to accelerate neurodegeneration [37,101,102]. Moreover, chronic oxidative stress can impair endothelial function by disrupting NO signalling pathways essential for vasodilation and maintaining CBF. The reaction between superoxide radicals and NO reduces its bioavailability; this reduction in NO not only impairs endothelial function but also disrupts intercellular signaling across the NV unit, particularly affecting neuron–astrocyte–vascular communication required for dynamic CBF regulation, leading to impaired vascular responses and contributing to further neurovascular dysfunction [103,104]. Taken together, these findings demonstrate that vascular dysfunction is not merely a consequence but a key contributor to Alzheimer’s disease progression.

## 4. NVU in PD

**Figure 2:**
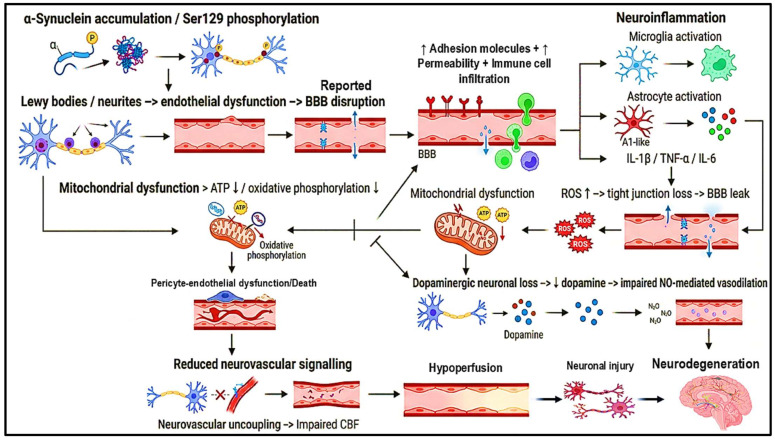
Proposed mechanisms of neurovascular uncoupling in Parkinson’s disease. This schematic represents how α-Synuclein accumulation, particularly Ser129-phosphorylated species, has been shown in experimental models to impair endothelial function and disrupt pericyte-endothelial interactions, contributing to BBB dysfunction [106,107,108,109,110,111]. In vitro and in vivo studies demonstrate that α-synuclein can induce endothelial activation, increase adhesion molecule expression, and promote barrier permeability [107,108]. Neuroinflammatory activation of microglia and astrocytes further exacerbates vascular dysfunction through cytokine release (e.g., TNF-α, IL-1β), which has been linked to BBB impairment in PD models [13,107,112,113]. Mitochondrial dysfunction contributes to endothelial injury via ROS generation and ATP depletion, leading to disruption of tight junction proteins, as demonstrated in toxin-induced and genetic PD models [114,115,116,117]. Additionally, dopaminergic neuronal loss may indirectly impair cerebrovascular regulation by disrupting NO-mediated vascular signaling pathways, although this mechanism is supported primarily by indirect and preclinical evidence [118,119,120]. Collectively, these pathways represent experimentally supported but not yet fully validated mechanisms of BBB dysfunction in PD. While multiple studies support the involvement of BBB dysfunction in PD, it is important to note that findings across clinical and experimental studies remain heterogeneous. Some neuroimaging studies report minimal or region-specific BBB leakage, whereas others suggest more widespread disruption depending on disease stage and model system. Furthermore, it remains unclear whether BBB dysfunction is an initiating factor or a downstream consequence of neurodegeneration. This distinction is critical, as many proposed mechanisms, including α-synuclein-mediated endothelial injury, mitochondrial dysfunction, and neuroinflammation, may independently contribute to both neuronal and vascular pathology. Therefore, future studies employing longitudinal imaging, standardized biomarkers, and human-based models are required to establish causality and clarify the precise role of BBB dysfunction in PD pathogenesis. Abbreviations: BBB: Blood–brain barrier; ROS: Reactive oxygen species; ATP: Adenosine triphosphate; NO: Nitric oxide; IL: Interleukin; TNF-α: Tumor necrosis factor-alpha; CBF: Cerebral blood flow.

PD-associated NVU is increasingly linked to vascular dysfunction, including BBB alterations; however, unlike AD, the causal and temporal role of BBB disruption in PD remains incompletely established. Current evidence primarily derives from experimental models, post-mortem analyses, and limited neuroimaging studies, which suggest associations rather than definitive causality [105]. Therefore, BBB dysfunction in PD should be interpreted as a contributing and potentially secondary phenomenon rather than a universally established primary driver. The mechanisms illustrated in Figure 2 represent proposed and disease-associated pathways supported by experimental and emerging clinical evidence but require further validation in longitudinal human studies (Figure 2).

### 4.1. α-Synuclein Aggregation and Vascular Dysfunction

PD is characterised by α-synuclein accumulation forming Lewy bodies/neurites, disrupting cellular homeostasis, impairing NV unit function, altering endothelial cells and pericytes, compromising BBB integrity, and promoting vascular dysfunction and neurodegeneration [14,15,121]. Phosphorylation of α-synuclein at Ser129 is a dominant pathological modification that enhances its aggregation propensity and contributes to vascular dysfunction, including BBB disruption and endothelial impairment [106]. Pathogenic α-synuclein has been reported to disrupt endothelial function, increase adhesion molecule expression, and enhance permeability in BBB models, particularly through inflammatory signaling pathways and oxidative stress mechanisms [13,107,108]. Initially, these models exhibit angiogenesis, an increase in new blood vessel formation. Vascular regression follows progression, shifting to pathology as toxic proteins accumulate. α-synuclein activates pericytes, disrupting BBB stability and CBF regulation via impaired interactions with endothelial cells and vascular smooth muscle cells [13,107,108]. This effect has been linked to pericyte-mediated BBB impairment in experimental models [12,109]. In PD models, pericytes show signs of pathological activation early in disease progression without a corresponding increase in pericyte density. This suggests that while the overall number of pericytes may remain stable, their functionality is compromised, leading to impaired regulation of blood flow and increased BBB permeability [109]. Moreover, α-synuclein aggregates can induce oxidative stress within endothelial cells. The presence of ROS generated by dysfunctional mitochondria contributes to cellular damage and exacerbates inflammation. This oxidative environment further destabilizes the BBB by disrupting tight junction proteins essential for maintaining endothelial cell cohesion. As a result, there is an increase in paracellular permeability, allowing for the leakage of plasma proteins into the brain parenchyma, which can lead to edema and neuronal injury [110,111].

### 4.2. Dopaminergic Neuronal Loss and Its Impact on Cerebrovascular Regulation

Degeneration of dopaminergic neurons, notably in the substantia nigra pars compacta, is a distinguishing hallmark of PD and has a significant impact on cerebrovascular control. The ensuing decrease in dopamine availability impairs both motor and cognitive control, while also jeopardising NV unit integrity. Beyond causing typical motor manifestations, including bradykinesia, stiffness, and tremor, dopaminergic neuronal loss disrupts neurovascular signalling, compromising coordinated CBF and vascular function [118,119,120]. Dopaminergic neurons regulate CBF via modulation of vascular tone, and experimental studies suggest that dopamine depletion may impair NO-mediated vasodilation and cerebrovascular responsiveness, although direct clinical evidence linking this mechanism to BBB dysfunction remains limited [120,122,123]. In the context of PD, this loss of dopaminergic regulation may contribute to impaired neurovascular responses shown in Figure 2 [118,119,120,122,123,124]. Dopaminergic signaling interacts with NO pathways to regulate vascular tone, and loss of dopamine in PD may indirectly impair NO-mediated vasodilation across NV unit components, further contributing to NVU. Research indicates that the initial stages of PD may involve synaptic dysfunction prior to significant neuronal death. Dysfunctional dopaminergic synapses can lead to neurotransmitter deficiencies that affect not only neuronal communication but also the signalling pathways involved in cerebrovascular regulation. This synaptic impairment can result in reduced NVC, where neuronal activity fails to elicit an appropriate increase in CBF. Consequently, this may contribute to hypoperfusion in certain brain regions, exacerbating neurodegeneration and affecting cognitive functions [15,124]. Dopaminergic neurons are energy-dependent due to branching and pacemaking activity; mitochondrial dysfunction and oxidative stress reduce ATP, impairing cellular functions and vascular regulation, decreasing their ability to modulate blood flow effectively [125]. The loss of dopaminergic neurons also impacts other neurotransmitter systems that are involved in cerebrovascular regulation. For instance, noradrenergic and serotonergic systems may become dysregulated due to the interconnected nature of these pathways with dopaminergic signalling. The interplay between these neurotransmitters is essential for maintaining vascular health; thus, their disruption can lead to additional cerebrovascular complications [125,126,127].

### 4.3. Mitochondrial Dysfunction and Oxidative Damage in NVU

In PD, mitochondrial dysfunction-induced oxidative damage is identified as a major contributor to neurovascular unit deterioration. Mitochondrial dysfunction in PD disrupts multiple NV unit components, impairing CBF and BBB integrity. In PD, mitochondrial dysfunction leads to significant metabolic alterations that result in increased oxidative stress, ultimately exacerbating neurodegeneration. Mitochondria are crucial for energy production, particularly in neurons that have high metabolic demands [114,115]. In PD, mitochondrial dysfunction is often characterized by impaired oxidative phosphorylation and reduced ATP production. This impairment can arise from genetic mutations associated with PD (such as those affecting PINK1 and Parkin), exposure to environmental toxins, or the accumulation of misfolded proteins like α-synuclein. The resultant energy deficit hampers the NV unit’s ability to regulate CBF effectively, leading to hypoperfusion and neuronal injury [128,129]. Mitochondrial dysfunction in PD is associated with excessive ROS generation, which has been experimentally shown to disrupt endothelial tight junction proteins such as claudin-5 and occludin, thereby contributing to BBB permeability in toxin-induced and genetic PD models [114,115,116,117,128,129]. This oxidative burden in the NV unit affects endothelial tight junction proteins such as claudin-5 and occludin, jeopardising the BBB integrity. The increased permeability allows neurotoxic substances to enter the brain parenchyma, boosting neuroinflammatory responses and accelerating neuronal damage [116,117].

Oxidative damage also affects pericyte contractile cells on capillaries that are vital for BBB integrity and CBF regulation. Under oxidative stress conditions, pericytes may undergo apoptosis or become dysfunctional. Studies indicate that ROS produced by activated microglia can induce pericyte death, further compromising BBB function. This loss of pericyte activity contributes to increased BBB permeability and neuronal injury. The relationship between mitochondrial dysfunction and neuroinflammation is particularly significant [130]. Mitochondrial damage can activate microglia, resulting in a pro-inflammatory response that perpetuates oxidative stress. Activated microglia release inflammatory cytokines and additional ROS, creating a damaging cycle that affects all components of the NV unit. This chronic neuroinflammatory state impairs vascular function and contributes to neuronal degeneration [110,131].

### 4.4. Role of Neuroinflammation in PD-Associated NVU

In PD, neuroinflammation arises primarily from the activation of microglia and astrocytes in response to pathological changes, particularly the aggregation of α-synuclein. Under normal conditions, microglia and astrocytes perform protective functions in the brain, such as releasing neurotrophic factors that support neuronal health [112]. Glial cells in PD undergo a reconfiguration from a protective to a pathogenic phenotype due to hereditary vulnerability and α-synuclein aggregation. Activated microglia and astrocytes generate pro-inflammatory mediators such as IL-1β, TNF-α, and IL-6, which may amplify endothelial dysfunction, increase barrier permeability, and exacerbate neurovascular impairment [112,113]. Importantly, this inflammatory cascade extends beyond the central nervous system, with new evidence linking systemic modulators, such as gut microbiota composition, to neuroinflammatory responses in PD [15,113].

The interaction between neuroinflammation and other pathological mechanisms in PD is complex [112]. For example, oxidative stress is often present alongside neuroinflammation, creating a detrimental cycle that further impairs the NV unit’s function. Inflammation-induced ROS generation contributes directly to endothelial damage, compromising BBB integrity. The increased barrier permeability allows peripheral immune cells to infiltrate the brain parenchyma, perpetuating inflammatory signalling and exacerbating neuronal injury [37]. Moreover, neuroinflammation affects dopaminergic neurons directly. The inflammatory milieu can enhance the intrinsic vulnerability of these neurons due to their unique metabolic requirements and high iron content. Factors such as altered calcium channel expression and a lower antioxidant defense system make dopaminergic neurons particularly susceptible to damage from inflammatory cytokines and ROS [37,121].

The role of neuroinflammation in PD is further complicated by its bidirectional relationship with mitochondrial dysfunction. Mitochondrial impairment can activate microglia, leading to increased production of inflammatory mediators. Conversely, chronic inflammation can exacerbate mitochondrial dysfunction, creating a vicious cycle that contributes to neuronal loss [112]. Research has shown that targeting neuroinflammatory pathways may offer therapeutic potential for PD. For instance, strategies aimed at modulating microglial activation or enhancing the production of anti-inflammatory cytokines could help restore balance within the NV units and protect against neuronal degeneration. Additionally, understanding how systemic factors influence neuroinflammation may lead to novel interventions that address both central and peripheral aspects of the disease [113,121]. Collectively, these mechanisms illustrate how neurovascular dysfunction contributes to both motor and non-motor features of Parkinson’s disease.

## 5. Comparative Insights: AD vs. PD

NVU in AD and PD is driven by several shared pathological mechanisms, including oxidative stress, neuroinflammation, endothelial dysfunction, BBB disruption, and reduced CBF [93,121,132]. These processes collectively impair vascular responsiveness and metabolic support, contributing to progressive neuronal dysfunction in both conditions. Despite these similarities, the upstream drivers of neurovascular impairment differ between the two diseases. In AD, amyloid-beta accumulation and tau pathology are the principal contributors to vascular dysfunction, promoting oxidative stress, endothelial injury, and impaired clearance mechanisms. In contrast, PD is primarily associated with alpha-synuclein aggregation, dopaminergic neuronal loss, and mitochondrial dysfunction, which together disrupt vascular regulation and enhance oxidative damage [16,133]. These disease-specific mechanisms lead to distinct patterns of neurovascular impairment. AD is more closely associated with early BBB breakdown and metabolic insufficiency, whereas PD demonstrates prominent mitochondrial dysfunction and altered vascular tone regulation. Understanding these differences is important for distinguishing disease progression and identifying targeted therapeutic strategies. Overall, while AD and PD converge on common neurovascular pathways, their distinct molecular origins shape differential vascular outcomes, highlighting the need for disease-specific approaches in diagnosis and treatment [118,119,134,135].

## 6. Imaging and Biomarker-Based Assessment of NV Unit

The assessment of the NV unit in neurological disorders such as AD and PD is crucial for understanding disease mechanisms and developing effective treatments. Advanced neuroimaging techniques, circulating biomarkers, and emerging technologies play significant roles in this assessment [136,137].

### 6.1. Advanced Neuroimaging Techniques for NVU Detection

Advances in neuroimaging technologies have significantly enhanced the in vivo evaluation of the NV unit. Functional magnetic resonance imaging (fMRI), diffusion tensor imaging, and arterial spin labelling (ASL) allow for a thorough assessment of both vascular dynamics and structural connections. fMRI allows for the analysis of CBF responses to neuronal activation, thereby assessing NVC, whereas diffusion tensor imaging characterises white matter integrity and network architecture, providing insights into the impact of vascular health on overall brain function [138,139]. Moreover, multi-modal imaging approaches are increasingly utilized to gain a comprehensive view of NVU. For instance, combining fMRI with ASL can reveal how changes in blood flow correlate with neuronal activity across different brain regions. Such techniques are particularly valuable in studying conditions like PD, where neurovascular decoupling has been associated with cognitive decline [140]. The integration of these advanced imaging modalities enhances our understanding of the pathophysiology underlying neurological disorders and aids in early diagnosis [141,142].

### 6.2. Circulating and Cerebrospinal Fluid (CSF) Biomarkers

In addition to imaging techniques, biomarkers found in blood and CSF are critical for assessing NV Unit integrity. Circulating biomarkers such as inflammatory cytokines, oxidative stress markers, and proteins associated with neurodegeneration can provide insights into the state of the NV Unit [143,144]. For example, elevated levels of certain cytokines may indicate ongoing neuroinflammation that affects vascular function. CSF biomarkers are particularly informative as they reflect changes occurring within the CNS more directly than peripheral markers. Biomarker selection should prioritise indicators that directly reflect NV Unit dysfunction, including endothelial injury, BBB permeability, and vascular inflammation [145,146].

### 6.3. Emerging Technologies for NVU Monitoring

Emerging technologies are poised to further enhance our ability to monitor the NV Unit. Innovations such as wearable devices that utilize near-infrared spectroscopy (NIRS) allow for continuous monitoring of cerebral oxygenation and blood flow in real-time [147]. This non-invasive approach can provide insights into how vascular health fluctuates during daily activities or in response to therapeutic interventions [148,149]. Recent advancements in molecular imaging have enabled in vivo visualisation of cellular and metabolic processes within the NV Unit. PET techniques allow for the measurement of neuroinflammatory activity as well as metabolic changes associated with neurodegeneration. Concurrently, the introduction of nanoparticle-based targeted delivery systems offers therapeutic potential by allowing for localised control of mitochondrial activity and oxidative stress reduction, consequently promoting neurovascular regeneration and protection [150] (Table 1).

## 7. Therapeutic Strategies Targeting NV Unit

The NV unit plays a crucial role in maintaining brain health and function, particularly in the context of neurodegenerative diseases such as AD and PD. Table 2 explores various therapeutic strategies targeting the NV Unit, including pharmacological interventions aimed at addressing endothelial dysfunction and modulating neuroinflammation, as well as non-pharmacological approaches like lifestyle modifications and advanced neuromodulatory techniques. By integrating these diverse strategies, we can enhance our understanding of disease mechanisms and improve patient outcomes in AD and PD [121,179]. Targeting tau phosphorylation and its downstream effects on NO signaling represents an emerging strategy for restoring neurovascular function in AD.

## 8. Future Directions

The future of NVU research in AD and PD is filled with promising advancements that may significantly enhance both our understanding and clinical management of these disorders. One of the most critical directions is the integration of personalized medicine, which combines genetic, epigenetic, and multi-omics data to create individualized therapeutic approaches [194]. The identification and validation of specific biomarkers for early detection of NVU are essential for effective disease management. Techniques such as liquid biopsy, which focus on circulating and cerebrospinal fluid biomarkers, will play a pivotal role in assessing NV dysfunction and tracking disease progression in real time. These biomarkers will be instrumental in providing tailored treatments for patients, optimizing therapeutic efficacy, and minimizing adverse effects. The advancement of neuroimaging techniques holds significant promise for future research. High-resolution MRI, positron emission tomography (PET), and functional imaging technologies are expected to improve the ability to visualize the NV dysfunction in vivo, particularly endothelial alterations, BBB integrity, and NVC in real-time. These imaging modalities could be utilized for early diagnosis, longitudinal monitoring, and evaluating the effects of novel therapeutic interventions aimed at restoring the NV unit’s function. Enhanced imaging capabilities will be instrumental in providing a more accurate assessment of the underlying neurovascular changes associated with AD and PD, thus paving the way for better-targeted treatments.

Future therapy development in Alzheimer’s and Parkinson’s diseases is likely to focus on new delivery platforms that can bypass or restore BBB malfunction. Nanoparticle-based systems and endothelial-targeted gene treatments show promise in improving brain-specific drug delivery, reducing neuroinflammation, maintaining barrier integrity, and restoring neurovascular signalling. In addition, pharmaceutical manipulation of endothelial pathways, such as VEGF and NO signalling, may improve NVC and decrease disease development. Furthermore, breakthroughs in stem cell-based and regenerative techniques have created new potential to heal vasculature and neural injury, providing complementary options for restoring neurovascular function. The potential use of endothelial progenitor cells or neural stem cells to repair or replace damaged endothelial cells and improve neurovascular function holds great promise. These stem cell-based approaches could contribute to restoring NVC and ameliorating the pathological consequences of NV dysfunction in both AD and PD. As research progresses, we expect these regenerative strategies to be refined, potentially leading to more effective treatments and improved clinical outcomes for patients with these debilitating neurodegenerative diseases.

## 9. Challenges and Opportunities in NVU Research

Translational progress in NVU research is hampered by numerous important impediments. Restricted BBB permeability impedes effective therapeutic administration, while the NV Unit’s intricate cellular architecture makes it difficult to identify precise molecular targets. Similarly, the lack of accurate biomarkers for early diagnosis and longitudinal surveillance causes delays in clinical action. Despite these limitations, developing multi-omics techniques that combine genomic, proteomic, and metabolomic data provide significant prospects for decoding NVU-associated molecular networks. These methods show promise in finding novel biomarkers and aiding the development of tailored therapeutics, ultimately enhancing precision medicine tactics for NVU-related neurodegenerative illnesses. These approaches also enable personalized medicine, optimizing treatment strategies. Collaboration between neuroscience and vascular biology is crucial to understanding the interplay between the brain’s vascular system and neural tissue. This cross-disciplinary effort can accelerate the development of targeted therapies, improve preclinical models, and enhance our understanding of NVU in neurodegenerative diseases like AD and PD. Through these combined efforts, the field is poised for significant advancements in both diagnostics and treatment.

## 10. Conclusions

NVU plays a critical role in the pathophysiology of AD and PD, contributing to neurodegeneration through disrupted BBB integrity, endothelial dysfunction, and neuroinflammation. Despite significant challenges, including the complexity of the BBB and the lack of effective biomarkers for early detection, recent advances in multi-omics technologies, neuroimaging, and drug delivery systems offer promising opportunities. The integration of these approaches could lead to better-targeted therapies and personalized medicine strategies, ultimately improving patient outcomes. Furthermore, interdisciplinary collaboration between neuroscience and vascular biology is essential to unravel the intricate mechanisms underlying NVU. As research continues to progress, there is a strong potential for new diagnostic tools and therapeutic interventions that could mitigate or even reverse the neurovascular dysfunction central to AD and PD.

## Figures and Tables

**Table 1:** Emerging Technologies for NVU Monitoring.

Assessment Technique	Modality/ Method	Biomarkers/ Indicators	Relevance in AD	Relevance in PD	References
Advanced Neuroimaging Techniques for NVU Detection	Dynamic Contrast-Enhanced MRI (DCE-MRI)	BBB permeability, vascular leakage	DCE-MRI detects abnormal BBB permeability in AD, revealing regions where NVU is prominent, often associated with amyloid plaques.	DCE-MRI in PD can highlight early BBB disruption in areas affected by dopaminergic loss, such as the substantia nigra.	[151,152]
	Arterial Spin Labeling (ASL)	CBF	ASL measures CBF, showing reduced perfusion and impaired NVC in AD, particularly in hippocampal and cortical regions.	ASL reveals CBF changes in PD, with reduced perfusion in basal ganglia and cortex correlating with motor and cognitive symptoms.	[153,154]
	Magnetic Resonance Spectroscopy (MRS)	Metabolites, including N-acetylaspartate (NAA), choline, creatine	MRS reveals metabolic shifts due to NVU, with decreased NAA levels indicating neuronal loss and impaired neurovascular function in AD.	MRS shows metabolic alterations in PD, such as reduced NAA in dopaminergic regions, which may be linked to neurovascular dysfunction.	[155,156]
	Functional MRI (fMRI)	Cortical activity, hemodynamic response	fMRI evaluates functional brain activity and BOLD response, showing impaired NVC and decreased brain response to stimuli in AD.	fMRI detects altered motor and cognitive function in PD, indicating disrupted NVC in dopaminergic regions.	[157,158]
	Diffusion Tensor Imaging (DTI)	White matter integrity, microstructural changes	DTI detects white matter degradation and microvascular changes due to NVU in AD, particularly in areas vulnerable to amyloid deposition.	DTI in PD reveals disruption of white matter tracts, particularly in the basal ganglia and prefrontal cortex, suggesting NVU.	[159,160]
	Positron Emission Tomography (PET)	Amyloid and tau burden, neuroinflammation, metabolic activity	PET imaging detects amyloid and tau deposition, offering insights into regions of impaired perfusion and neurovascular dysfunction.	PET imaging in PD assesses neuroinflammation and metabolic changes in dopaminergic regions, indicating early NVU and neurodegeneration.	[161,162]
Circulating and CSF Biomarkers	CSF Analysis (Aβ42, tau, p-tau, and neuroinflammatory markers)	Aβ42, tau, p-tau, neuroinflammatory cytokines (e.g., IL-6, TNF-α)	CSF levels of Aβ42 and tau correlate with BBB dysfunction, indicating vascular changes and neuroinflammation in AD. Elevated tau and p-tau suggest neuronal injury and disrupted NVC.	Elevated CSF levels of inflammatory markers such as IL-6 and TNF-α in PD reflect neuroinflammation and BBB disruption, indicative of NVU.	[143,163]
	Circulating MicroRNAs (miRNAs)	miRNA expression profiles (e.g., miR-132, miR-155)	Circulating miRNAs, particularly miR-132 and miR-155, are linked to vascular dysfunction and neuroinflammation in AD. These miRNAs are potential biomarkers for early NVU detection.	In PD, circulating miRNAs (e.g., miR-132) may serve as biomarkers of neuroinflammation and vascular changes in the early stages of disease.	[164,165]
	Circulating endothelial biomarkers (e.g., von Willebrand factor, sE-selectin)	Endothelial cell dysfunction markers	Elevated von Willebrand factor (vWF) and sE-selectin in AD indicate endothelial damage and disrupted BBB integrity, reflecting NVU.	In PD, circulating endothelial biomarkers, such as vWF, are elevated, reflecting endothelial dysfunction and BBB compromise in dopaminergic regions.	[166,167]
	Plasma Aβ and Tau levels	Aβ and tau levels in plasma	Elevated plasma Aβ and tau levels reflect the degree of NV dysfunction and Aβ accumulation in AD. These can be used as non-invasive biomarkers for NVU.	In PD, elevated plasma tau levels may indicate neurodegeneration and BBB disruption, potentially serving as biomarkers for NVU in early stages.	[168,169]
	Soluble Vascular Cell Adhesion Molecule-1 (sVCAM-1)	Endothelial activation and BBB permeability markers	Elevated sVCAM-1 in AD is associated with endothelial activation, neuroinflammation, and BBB leakage, contributing to NVU.	PD patients show increased levels of sVCAM-1, correlating with neuroinflammation and endothelial dysfunction in the BBB.	[170,171]
Emerging Technologies for NVU Monitoring	Optical Coherence Tomography (OCT)	Retinal microvascular changes	OCT and OCT angiography (OCTA) detect retinal vascular changes, which reflect neurovascular dysfunction in AD. Retinal microangiopathy is linked to BBB breakdown and cognitive decline.	OCTA can detect early signs of vascular dysfunction in the retina, reflecting changes in the brain’s vasculature in PD patients.	[172]
	Near-Infrared Spectroscopy (NIRS)	Cerebral oxygenation, perfusion, and hemodynamics	NIRS non-invasively measures cerebral oxygenation and perfusion, detecting impaired NVC in AD.	NIRS in PD provides real-time assessment of cerebral oxygenation and blood flow, aiding in the identification of NVU-related deficits.	[173,174]
	Wearable Devices for Cerebral Hemodynamics (e.g., transcranial Doppler Ultrasound)	CBF velocity	Wearable devices using Doppler ultrasound assess real-time CBF changes, offering insights into NVC and NVU in AD and PD.	Wearable Doppler devices in PD track changes in CBF velocity, which is useful for monitoring neurovascular health and identifying areas of dysfunction.	[175,176]
	Bioluminescent Imaging	In vivo detection of neuroinflammation and vascular changes	Bioluminescent imaging allows monitoring of neuroinflammation in AD models and offers insights into vascular changes and BBB breakdown.	In PD, bioluminescence can track the progression of inflammation and BBB permeability, providing real-time NVU monitoring in animal models.	[177,178]

**Table 2:** Therapeutic Strategies Targeting NV Unit.

Therapeutic Strategy	Intervention Type	Mechanism/ Target	Relevance in AD	Relevance in PD	References
Pharmacological Interventions	Drugs Targeting Endothelial Dysfunction	Drugs that protect endothelial cells and restore BBB integrity.	Statins (e.g., atorvastatin) and ARBs (e.g., losartan) have demonstrated BBB protective effects in AD models, reducing amyloid-related vascular damage.	ACE inhibitors (e.g., ramipril) and ARBs have shown promise in PD, helping to preserve BBB integrity and reduce vascular-related neurodegeneration.	[180,181]
	Agents Modulating Neuroinflammation and Oxidative Stress	Anti-inflammatory and antioxidant agents to reduce neuroinflammation and oxidative stress associated with NV Unit.	NSAIDs (e.g., ibuprofen) have been studied for their potential to mitigate neuroinflammation and BBB disruption in AD, though clinical efficacy remains debated. Curcumin and minocycline are promising adjunctive therapies for reducing oxidative stress and inflammation.	In PD, antioxidants like CoQ10, and anti-inflammatory agents like minocycline, may reduce oxidative damage and neuroinflammation, supporting vascular health in PD.	[37,182]
Non-Pharmacological Approaches	Lifestyle Interventions (Exercise, Diet)	Lifestyle changes aimed at improving cerebral circulation, reducing inflammation, and enhancing NVC.	Aerobic exercise has been shown to enhance neurovascular function, promote angiogenesis, and reduce amyloid plaque burden in AD models. The Mediterranean diet (rich in antioxidants and omega-3 fatty acids) has been shown to protect against neurovascular dysfunction.	Exercise and diet, including the Mediterranean diet and caloric restriction, improve NVC, cerebral perfusion, and dopaminergic function in PD. Regular physical activity is associated with slower cognitive decline and reduced neuroinflammation.	[183,184,185]
	Advanced Neuromodulatory Techniques	Use of brain stimulation methods to enhance NVC and cognitive function.	Transcranial magnetic stimulation (TMS) has been explored in AD to enhance NVC and reduce cognitive decline. Additionally, transcranial direct current stimulation (tDCS) has shown positive effects on cognitive performance by improving cortical excitability and brain perfusion.	In PD, DBS of the subthalamic nucleus and tDCS have been studied for their potential to alleviate motor symptoms, enhance neurovascular function, and improve cognitive aspects by improving brain perfusion and neuronal connectivity.	[186,187,188]
Emerging and Combination Therapies	Targeted Drug Delivery (Nanomedicine)	Nanocarriers for targeted delivery to the BBB.	Nanoparticle-based drug delivery systems targeting amyloid plaques and improving endothelial function hold promise in AD for delivering anti-inflammatory and anti-amyloid agents across the BBB.	Nanomedicines can be used to deliver neuroprotective agents such as antioxidants and anti-inflammatory drugs directly to the brain in PD, bypassing BBB disruption and improving neurovascular integrity.	[189,190]
	Stem Cell Therapy	Neural and endothelial progenitor cells to repair damaged NV units.	Stem cells (e.g., endothelial progenitor cells) have been tested in AD to promote angiogenesis and restore endothelial function in the cerebral vasculature, potentially reversing aspects of NVU.	Stem cell-based approaches, such as dopaminergic neuron replacement or endothelial progenitor cell transplantation, aim to restore vascular integrity and dopaminergic function in PD.	[191,192]
	Gene Therapy for Endothelial Dysfunction	Gene delivery to repair or enhance endothelial cell function.	Gene therapies targeting eNOS and VEGF expression show promise in improving BBB integrity and function in AD models.	Gene therapy in PD aims to restore neurovascular integrity by upregulating VEGF or eNOS in the basal ganglia to improve cerebral perfusion and reduce neuroinflammation.	[19,193]

## Data Availability

No new data were created or analyzed in this study. Data sharing is not applicable to this article.

## Abbreviations

Aβ	Amyloid-β
ACE	Angiotensin-converting enzyme
AD	Alzheimer’s disease
ASL	Arterial spin labeling
ATP	Adenosine triphosphate
ApoE	Apolipoprotein E
ApoE4	Apolipoprotein E4
BBB	Blood–brain barrier
BOLD	Blood oxygen level-dependent
CBF	Cerebral blood flow
CDK5	Cyclin-dependent kinase 5
CNS	Central nervous system
CSF	Cerebrospinal fluid
DCE-MRI	Dynamic contrast-enhanced magnetic resonance imaging
DBS	Deep brain stimulation
DTI	Diffusion tensor imaging
ECM	Extracellular matrix
eNOS	Endothelial nitric oxide synthase
ERK1/2	Extracellular signal-regulated kinase 1/2
fMRI	Functional magnetic resonance imaging
GLUT1	Glucose transporter 1
GSNO	S-nitrosoglutathione
IL	Interleukin
iNOS	Inducible nitric oxide synthase
JNK	c-Jun N-terminal kinase
LRP1	Low-density lipoprotein receptor-related protein 1
MAPK	Mitogen-activated protein kinase
MMPs	Matrix metalloproteinases
MRI	Magnetic resonance imaging
MRS	Magnetic resonance spectroscopy
NAA	N-acetylaspartate
NF-κB	Nuclear factor kappa B
NIRS	Near-infrared spectroscopy
NO	Nitric oxide
nNOS	Neuronal nitric oxide synthase
NVC	Neurovascular coupling
NV unit	Neurovascular unit
NVU	Neurovascular uncoupling
OCT	Optical coherence tomography
OCTA	Optical coherence tomography angiography
PD	Parkinson’s disease
PET	Positron emission tomography
PHF-1	Paired helical filament-1
PI3K	Phosphoinositide 3-kinase
RAGE	Receptor for advanced glycation end products
ROS	Reactive oxygen species
sGC	Soluble guanylate cyclase
sVCAM-1	Soluble vascular cell adhesion molecule-1
TGF-β	Transforming growth factor-beta
TMS	Transcranial magnetic stimulation
tDCS	Transcranial direct current stimulation
TNF-α	Tumor necrosis factor-alpha
VEGF	Vascular endothelial growth factor
VEGFR	Vascular endothelial growth factor receptor
vWF	von Willebrand factor
ZO-1	Zonula occludens-1

## References

[1] Negri S., Faris P., Soda T., Moccia F. (2021). Endothelial Signaling at the Core of Neurovascular Coupling: The Emerging Role of Endothelial Inward-Rectifier K+ (Kir2.1) Channels and N-Methyl-d-Aspartate Receptors in the Regulation of Cerebral Blood Flow. Int. J. Biochem. Cell Biol..

[2] Zhu W.M., Neuhaus A., Beard D.J., Sutherland B.A., DeLuca G.C. (2022). Neurovascular Coupling Mechanisms in Health and Neurovascular Uncoupling in Alzheimer’s Disease. Brain.

[3] Yu X., Ji C., Shao A. (2020). Neurovascular Unit Dysfunction and Neurodegenerative Disorders. Front. Neurosci..

[4] Kisler K., Nelson A.R., Montagne A., Zlokovic B.V. (2017). Cerebral Blood Flow Regulation and Neurovascular Dysfunction in Alzheimer Disease. Nat. Rev. Neurosci..

[5] Sweeney M.D., Kisler K., Montagne A., Toga A.W., Zlokovic B.V. (2018). The Role of Brain Vasculature in Neurodegenerative Disorders. Nat. Neurosci..

[6] Braun M., Iliff J.J. (2020). The Impact of Neurovascular, Blood-Brain Barrier, and Glymphatic Dysfunction in Neurodegenerative and Metabolic Diseases. Int. Rev. Neurobiol..

[7] Kunz A., Iadecola C. (2008). Chapter 14 Cerebral Vascular Dysregulation in the Ischemic Brain. Handbook of Clinical Neurology.

[8] Mena M.A., Rodríguez-Navarro J.A., de Yébenes J.G. (2009). The Multiple Mechanisms of Amyloid Deposition: The Role of Parkin. Prion.

[9] Abyadeh M., Gupta V., Paulo J.A., Mahmoudabad A.G., Shadfar S., Mirshahvaladi S., Gupta V., Nguyen C.T.O., Finkelstein D.I., You Y. (2024). Amyloid-Beta and Tau Protein beyond Alzheimer’s Disease. Neural Regen. Res..

[10] Garbuz D.G., Zatsepina O.G., Evgen’ev M.B. (2021). Beta Amyloid, Tau Protein, and Neuroinflammation: An Attempt to Integrate Different Hypotheses of Alzheimer’s Disease Pathogenesis. Mol. Biol..

[11] Canepa E., Fossati S. (2021). Impact of Tau on Neurovascular Pathology in Alzheimer’s Disease. Front. Neurol..

[12] Dohgu S., Takata F., Matsumoto J., Kimura I., Yamauchi A., Kataoka Y. (2019). Monomeric α-Synuclein Induces Blood–Brain Barrier Dysfunction through Activated Brain Pericytes Releasing Inflammatory Mediators in Vitro. Microvasc. Res..

[13] Albert K., Kälvälä S., Hakosalo V., Syvänen V., Krupa P., Niskanen J., Peltonen S., Sonninen T.-M., Lehtonen Š (2022). Cellular Models of Alpha-Synuclein Aggregation: What Have We Learned and Implications for Future Study. Biomedicines.

[14] Mehra S., Sahay S., Maji S.K. (2019). α-Synuclein Misfolding and Aggregation: Implications in Parkinson’s Disease Pathogenesis. Biochim. Biophys. Acta (BBA) Proteins Proteom..

[15] Calabresi P., Mechelli A., Natale G., Volpicelli-Daley L., Di Lazzaro G., Ghiglieri V. (2023). Alpha-Synuclein in Parkinson’s Disease and Other Synucleinopathies: From Overt Neurodegeneration Back to Early Synaptic Dysfunction. Cell Death Dis..

[16] Lei T., Yang Z., Li H., Qin M., Gao H. (2024). Interactions between Nanoparticles and Pathological Changes of Vascular in Alzheimer’s Disease. Adv. Drug Deliv. Rev..

[17] Stanimirovic D.B., Friedman A. (2012). Pathophysiology of the Neurovascular Unit: Disease Cause or Consequence?. J. Cereb. Blood Flow Metab..

[18] Wang L., Xiong X., Zhang L., Shen J. (2021). Neurovascular Unit: A Critical Role in Ischemic Stroke. CNS Neurosci. Ther..

[19] Su J.B. (2015). Vascular Endothelial Dysfunction and Pharmacological Treatment. World J. Cardiol..

[20] Wang X., He B. (2024). Endothelial Dysfunction: Molecular Mechanisms and Clinical Implications. MedComm.

[21] Meza C.A., La Favor J.D., Kim D.-H., Hickner R.C. (2019). Endothelial Dysfunction: Is There a Hyperglycemia-Induced Imbalance of NOX and NOS?. Int. J. Mol. Sci..

[22] O’Gallagher K., Rosentreter R.E., Elaine Soriano J., Roomi A., Saleem S., Lam T., Roy R., Gordon G.R., Raj S.R., Chowienczyk P.J. (2022). The Effect of a Neuronal Nitric Oxide Synthase Inhibitor on Neurovascular Regulation in Humans. Circ. Res..

[23] Takano T., Tian G.-F., Peng W., Lou N., Libionka W., Han X., Nedergaard M. (2006). Astrocyte-Mediated Control of Cerebral Blood Flow. Nat. Neurosci..

[24] Tran C.H.T., Peringod G., Gordon G.R. (2018). Astrocytes Integrate Behavioral State and Vascular Signals during Functional Hyperemia. Neuron.

[25] Liu C.-Y., Yang Y., Ju W.-N., Wang X., Zhang H.-L. (2018). Emerging Roles of Astrocytes in Neuro-Vascular Unit and the Tripartite Synapse With Emphasis on Reactive Gliosis in the Context of Alzheimer’s Disease. Front. Cell. Neurosci..

[26] Manu D.R., Slevin M., Barcutean L., Forro T., Boghitoiu T., Balasa R. (2023). Astrocyte Involvement in Blood–Brain Barrier Function: A Critical Update Highlighting Novel, Complex, Neurovascular Interactions. Int. J. Mol. Sci..

[27] He L., Zhang R., Yang M., Lu M. (2024). The Role of Astrocyte in Neuroinflammation in Traumatic Brain Injury. Biochim. Biophys. Acta (BBA) Mol. Basis Dis..

[28] de Rus Jacquet A., Alpaugh M., Denis H.L., Tancredi J.L., Boutin M., Decaestecker J., Beauparlant C., Herrmann L., Saint-Pierre M., Parent M. (2023). The Contribution of Inflammatory Astrocytes to BBB Impairments in a Brain-Chip Model of Parkinson’s Disease. Nat. Commun..

[29] Kempuraj D., Dourvetakis K.D., Cohen J., Valladares D.S., Joshi R.S., Kothuru S.P., Anderson T., Chinnappan B., Cheema A.K., Klimas N.G. (2024). Neurovascular Unit, Neuroinflammation and Neurodegeneration Markers in Brain Disorders. Front. Cell. Neurosci..

[30] Yan C., Zhou Y., Chen Q., Luo Y., Zhang J.H., Huang H., Shao A. (2020). Dysfunction of the Neurovascular Unit in Diabetes-Related Neurodegeneration. Biomed. Pharmacother..

[31] Li C., Wang Y., Yan X., Guo Z., Yang Y. (2021). Pathological Changes in Neurovascular Units: Lessons from Cases of Vascular Dementia. CNS Neurosci. Ther..

[32] Zhang J., Xie D., Jiao D., Zhou S., Liu S., Ju Z., Hu L., Qi L., Yao C., Zhao C. (2024). From Inflammatory Signaling to Neuronal Damage: Exploring NLR Inflammasomes in Ageing Neurological Disorders. Heliyon.

[33] Thurgur H., Pinteaux E. (2019). Microglia in the Neurovascular Unit: Blood–Brain Barrier–Microglia Interactions After Central Nervous System Disorders. Neuroscience.

[34] Takata F., Nakagawa S., Matsumoto J., Dohgu S. (2021). Blood-Brain Barrier Dysfunction Amplifies the Development of Neuroinflammation: Understanding of Cellular Events in Brain Microvascular Endothelial Cells for Prevention and Treatment of BBB Dysfunction. Front. Cell. Neurosci..

[35] Shibuya M. (2011). Vascular Endothelial Growth Factor (VEGF) and Its Receptor (VEGFR) Signaling in Angiogenesis: A Crucial Target for Anti- and Pro-Angiogenic Therapies. Genes Cancer.

[36] Rinaldi C., Donato L., Alibrandi S., Scimone C., D’Angelo R., Sidoti A. (2021). Oxidative Stress and the Neurovascular Unit. Life.

[37] Dash U.C., Bhol N.K., Swain S.K., Samal R.R., Nayak P.K., Raina V., Panda S.K., Kerry R.G., Duttaroy A.K., Jena A.B. (2024). Oxidative Stress and Inflammation in the Pathogenesis of Neurological Disorders: Mechanisms and Implications. Acta Pharm. Sin. B.

[38] Peng L., Zhang Z., Li Q., Song Z., Yan C., Ling H. (2024). Unveiling the Multifaceted Pathogenesis and Therapeutic Drugs of Alzheimer’s Disease: A Comprehensive Review. Heliyon.

[39] Solis E., Hascup K.N., Hascup E.R. (2020). Alzheimer’s Disease: The Link Between Amyloid-β and Neurovascular Dysfunction. J. Alzheimer’s Dis..

[40] Hampel H., Hardy J., Blennow K., Chen C., Perry G., Kim S.H., Villemagne V.L., Aisen P., Vendruscolo M., Iwatsubo T. (2021). The Amyloid-β Pathway in Alzheimer’s Disease. Mol. Psychiatry.

[41] Smith E.E., Greenberg S.M. (2009). β-Amyloid, Blood Vessels, and Brain Function. Stroke.

[42] Ma Q., Zhao Z., Sagare A.P., Wu Y., Wang M., Owens N.C., Verghese P.B., Herz J., Holtzman D.M., Zlokovic B.V. (2018). Blood-Brain Barrier-Associated Pericytes Internalize and Clear Aggregated Amyloid-Β42 by LRP1-Dependent Apolipoprotein E Isoform-Specific Mechanism. Mol. Neurodegener..

[43] Wang D., Chen F., Han Z., Yin Z., Ge X., Lei P. (2021). Relationship Between Amyloid-β Deposition and Blood–Brain Barrier Dysfunction in Alzheimer’s Disease. Front. Cell. Neurosci..

[44] MacVicar B.A., Newman E.A. (2015). Astrocyte Regulation of Blood Flow in the Brain. Cold Spring Harb. Perspect. Biol..

[45] Ding Z.-B., Song L.-J., Wang Q., Kumar G., Yan Y.-Q., Ma C.-G. (2021). Astrocytes: A Double-Edged Sword in Neurodegenerative Diseases. Neural Regen. Res..

[46] Christie R., Yamada M., Moskowitz M., Hyman B. (2001). Structural and Functional Disruption of Vascular Smooth Muscle Cells in a Transgenic Mouse Model of Amyloid Angiopathy. Am. J. Pathol..

[47] Cheung C., Goh Y.T., Zhang J., Wu C., Guccione E. (2014). Modeling Cerebrovascular Pathophysiology in Amyloid-β Metabolism Using Neural-Crest-Derived Smooth Muscle Cells. Cell Rep..

[48] Yue Q., Song Y., Liu Z., Zhang L., Yang L., Li J. (2022). Receptor for Advanced Glycation End Products (RAGE): A Pivotal Hub in Immune Diseases. Molecules.

[49] Kierdorf K., Fritz G. (2013). RAGE Regulation and Signaling in Inflammation and Beyond. J. Leukoc. Biol..

[50] Zhang H., Jiang X., Ma L., Wei W., Li Z., Chang S., Wen J., Sun J., Li H. (2022). Role of Aβ in Alzheimer’s-Related Synaptic Dysfunction. Front. Cell Dev. Biol..

[51] Ricciarelli R., Fedele E. (2017). The Amyloid Cascade Hypothesis in Alzheimer’s Disease: It’s Time to Change Our Mind. Curr. Neuropharmacol..

[52] Barage S.H., Sonawane K.D. (2015). Amyloid Cascade Hypothesis: Pathogenesis and Therapeutic Strategies in Alzheimer’s Disease. Neuropeptides.

[53] Park L., Hochrainer K., Hattori Y., Ahn S.J., Anfray A., Wang G., Uekawa K., Seo J., Palfini V., Blanco I. (2020). Tau Induces PSD95–Neuronal NOS Uncoupling and Neurovascular Dysfunction Independent of Neurodegeneration. Nat. Neurosci..

[54] Hussong S.A., Banh A.Q., Van Skike C.E., Dorigatti A.O., Hernandez S.F., Hart M.J., Ferran B., Makhlouf H., Gaczynska M., Osmulski P.A. (2023). Soluble Pathogenic Tau Enters Brain Vascular Endothelial Cells and Drives Cellular Senescence and Brain Microvascular Dysfunction in a Mouse Model of Tauopathy. Nat. Commun..

[55] Rawat P., Sehar U., Bisht J., Selman A., Culberson J., Reddy P.H. (2022). Phosphorylated Tau in Alzheimer’s Disease and Other Tauopathies. Int. J. Mol. Sci..

[56] Medeiros R., Baglietto-Vargas D., LaFerla F.M. (2011). The Role of Tau in Alzheimer’s Disease and Related Disorders. CNS Neurosci. Ther..

[57] Parra Bravo C., Naguib S.A., Gan L. (2024). Cellular and Pathological Functions of Tau. Nat. Rev. Mol. Cell Biol..

[58] Reddy P.H. (2011). Abnormal Tau, Mitochondrial Dysfunction, Impaired Axonal Transport of Mitochondria, and Synaptic Deprivation in Alzheimer’s Disease. Brain Res..

[59] Brunello C.A., Merezhko M., Uronen R.-L., Huttunen H.J. (2020). Mechanisms of Secretion and Spreading of Pathological Tau Protein. Cell. Mol. Life Sci..

[60] Wang P., Ye Y. (2021). Filamentous Recombinant Human Tau Activates Primary Astrocytes via an Integrin Receptor Complex. Nat. Commun..

[61] Maté de Gérando A., d’Orange M., Augustin E., Joséphine C., Aurégan G., Gaudin-Guérif M., Guillermier M., Hérard A.-S., Stimmer L., Petit F. (2021). Neuronal Tau Species Transfer to Astrocytes and Induce Their Loss According to Tau Aggregation State. Brain.

[62] Singh D. (2022). Astrocytic and Microglial Cells as the Modulators of Neuroinflammation in Alzheimer’s Disease. J. Neuroinflamm..

[63] Sengupta A., Kabat J., Novak M., Wu Q., Grundke-Iqbal I., Iqbal K. (1998). Phosphorylation of Tau at Both Thr 231 and Ser 262 Is Required for Maximal Inhibition of Its Binding to Microtubules. Arch. Biochem. Biophys..

[64] Chen J., Ye Z., Wang X., Chang J., Yang M., Zhong H., Hong F., Yang S. (2018). Nitric Oxide Bioavailability Dysfunction Involves in Atherosclerosis. Biomed. Pharmacother..

[65] Yang Y., Torbey M.T. (2020). Angiogenesis and Blood-Brain Barrier Permeability in Vascular Remodeling after Stroke. Curr. Neuropharmacol..

[66] Benarroch E. (2023). What Are the Roles of Pericytes in the Neurovascular Unit and Its Disorders?. Neurology.

[67] Sweeney M.D., Ayyadurai S., Zlokovic B.V. (2016). Pericytes of the Neurovascular Unit: Key Functions and Signaling Pathways. Nat. Neurosci..

[68] ElAli A., Thériault P., Rivest S. (2014). The Role of Pericytes in Neurovascular Unit Remodeling in Brain Disorders. Int. J. Mol. Sci..

[69] Ojo J., Eisenbaum M., Shackleton B., Lynch C., Joshi U., Saltiel N., Pearson A., Ringland C., Paris D., Mouzon B. (2021). Mural Cell Dysfunction Leads to Altered Cerebrovascular Tau Uptake Following Repetitive Head Trauma. Neurobiol. Dis..

[70] Austin S.A., Katusic Z.S. (2016). Loss of Endothelial Nitric Oxide Synthase Promotes P25 Generation and Tau Phosphorylation in a Murine Model of Alzheimer’s Disease. Circ. Res..

[71] Won J.-S., Annamalai B., Choi S., Singh I., Singh A.K. (2015). S-Nitrosoglutathione Reduces Tau Hyper-Phosphorylation and Provides Neuroprotection in Rat Model of Chronic Cerebral Hypoperfusion. Brain Res..

[72] Zhang Y.-J., Xu Y.-F., Liu Y.-H., Yin J., Wang J.-Z. (2005). Nitric Oxide Induces Tau Hyperphosphorylation via Glycogen Synthase Kinase-3β Activation. FEBS Lett..

[73] Španić E., Langer Horvat L., Hof P.R., Šimić G. (2019). Role of Microglial Cells in Alzheimer’s Disease Tau Propagation. Front. Aging Neurosci..

[74] Jin M., Shiwaku H., Tanaka H., Obita T., Ohuchi S., Yoshioka Y., Jin X., Kondo K., Fujita K., Homma H. (2021). Tau Activates Microglia via the PQBP1-CGAS-STING Pathway to Promote Brain Inflammation. Nat. Commun..

[75] Woodburn S.C., Bollinger J.L., Wohleb E.S. (2021). The Semantics of Microglia Activation: Neuroinflammation, Homeostasis, and Stress. J. Neuroinflamm..

[76] Wu Y.-C., Bogale T.A., Koistinaho J., Pizzi M., Rolova T., Bellucci A. (2024). The Contribution of β-Amyloid, Tau and α-Synuclein to Blood–Brain Barrier Damage in Neurodegenerative Disorders. Acta Neuropathol..

[77] Vogels T., Murgoci A.-N., Hromádka T. (2019). Intersection of Pathological Tau and Microglia at the Synapse. Acta Neuropathol. Commun..

[78] Rather M.A., Khan A., Jahan S., Siddiqui A.J., Wang L. (2024). Influence of Tau on Neurotoxicity and Cerebral Vasculature Impairment Associated with Alzheimer’s Disease. Neuroscience.

[79] Sato H., Kato T., Arawaka S. (2013). The Role of Ser129 Phosphorylation of α-Synuclein in Neurodegeneration of Parkinson’s Disease: A Review of in Vivo Models. Rev. Neurosci..

[80] Yokoya M., Takata F., Iwao T., Matsumoto J., Tanaka Y., Aridome H., Yasunaga M., Mizoguchi J., Sano K., Dohgu S. (2025). α-Synuclein Degradation in Brain Pericytes Is Mediated via Akt, ERK, and P38 MAPK Signaling Pathways. Int. J. Mol. Sci..

[81] Yuan Y., Sun J., Dong Q., Cui M. (2023). Blood–Brain Barrier Endothelial Cells in Neurodegenerative Diseases: Signals from the “Barrier”. Front. Neurosci..

[82] Archie S.R., Al Shoyaib A., Cucullo L. (2021). Blood-Brain Barrier Dysfunction in CNS Disorders and Putative Therapeutic Targets: An Overview. Pharmaceutics.

[83] Huang Z., Wong L.-W., Su Y., Huang X., Wang N., Chen H., Yi C. (2020). Blood-Brain Barrier Integrity in the Pathogenesis of Alzheimer’s Disease. Front. Neuroendocrinol..

[84] Tao Q.-Q., Lin R.-R., Chen Y.-H., Wu Z.-Y. (2022). Discerning the Role of Blood Brain Barrier Dysfunction in Alzheimer’s Disease. Aging Dis..

[85] Jiang X., Andjelkovic A.V., Zhu L., Yang T., Bennett M.V.L., Chen J., Keep R.F., Shi Y. (2018). Blood-Brain Barrier Dysfunction and Recovery after Ischemic Stroke. Prog. Neurobiol..

[86] Cerutti C., Ridley A.J. (2017). Endothelial Cell-Cell Adhesion and Signaling. Exp. Cell Res..

[87] van Hinsbergh V.W.M. (2012). Endothelium—Role in Regulation of Coagulation and Inflammation. Semin. Immunopathol..

[88] Chen Y., Joo J., Chu J.M.-T., Chang R.C.-C., Wong G.T.-C. (2023). Downregulation of the Glucose Transporter GLUT 1 in the Cerebral Microvasculature Contributes to Postoperative Neurocognitive Disorders in Aged Mice. J. Neuroinflamm..

[89] Głuchowska K., Pliszka M., Szablewski L. (2021). Expression of Glucose Transporters in Human Neurodegenerative Diseases. Biochem. Biophys. Res. Commun..

[90] Simpson I.A., Chundu K.R., Davies-Hill T., Honer W.G., Davies P. (1994). Decreased Concentrations of GLUT1 and GLUT3 Glucose Transporters in the Brains of Patients with Alzheimer’s Disease. Ann. Neurol..

[91] Winkler E.A., Nishida Y., Sagare A.P., Rege S.V., Bell R.D., Perlmutter D., Sengillo J.D., Hillman S., Kong P., Nelson A.R. (2015). GLUT1 Reductions Exacerbate Alzheimer’s Disease Vasculo-Neuronal Dysfunction and Degeneration. Nat. Neurosci..

[92] Parhizkar S., Holtzman D.M. (2022). APOE Mediated Neuroinflammation and Neurodegeneration in Alzheimer’s Disease. Semin. Immunol..

[93] Kinney J.W., Bemiller S.M., Murtishaw A.S., Leisgang A.M., Salazar A.M., Lamb B.T. (2018). Inflammation as a Central Mechanism in Alzheimer’s Disease. Alzheimer’s Dement. Transl. Res. Clin. Interv..

[94] Lee R., Channon K.M., Antoniades C. (2012). Therapeutic Strategies Targeting Endothelial Function in Humans:Clinical Implications. Curr. Vasc. Pharmacol..

[95] Wang L., Cheng C.K., Yi M., Lui K.O., Huang Y. (2022). Targeting Endothelial Dysfunction and Inflammation. J. Mol. Cell. Cardiol..

[96] Krylatov A.V., Maslov L.N., Voronkov N.S., Boshchenko A.A., Popov S.V., Gomez L., Wang H., Jaggi A.S., Downey J.M. (2018). Reactive Oxygen Species as Intracellular Signaling Molecules in the Cardiovascular System. Curr. Cardiol. Rev..

[97] Kim G.W., Gasche Y., Grzeschik S., Copin J.-C., Maier C.M., Chan P.H. (2003). Neurodegeneration in Striatum Induced by the Mitochondrial Toxin 3-Nitropropionic Acid: Role of Matrix Metalloproteinase-9 in Early Blood-Brain Barrier Disruption?. J. Neurosci..

[98] Pizzino G., Irrera N., Cucinotta M., Pallio G., Mannino F., Arcoraci V., Squadrito F., Altavilla D., Bitto A. (2017). Oxidative Stress: Harms and Benefits for Human Health. Oxid. Med. Cell. Longev..

[99] Hong Y., Boiti A., Vallone D., Foulkes N.S. (2024). Reactive Oxygen Species Signaling and Oxidative Stress: Transcriptional Regulation and Evolution. Antioxidants.

[100] Teleanu D.M., Niculescu A.-G., Lungu I.I., Radu C.I., Vladâcenco O., Roza E., Costăchescu B., Grumezescu A.M., Teleanu R.I. (2022). An Overview of Oxidative Stress, Neuroinflammation, and Neurodegenerative Diseases. Int. J. Mol. Sci..

[101] Chatterjee S. (2016). Oxidative Stress, Inflammation, and Disease. Oxidative Stress and Biomaterials.

[102] Mittal M., Siddiqui M.R., Tran K., Reddy S.P., Malik A.B. (2014). Reactive Oxygen Species in Inflammation and Tissue Injury. Antioxid. Redox Signal..

[103] Higashi Y., Maruhashi T., Noma K., Kihara Y. (2014). Oxidative Stress and Endothelial Dysfunction: Clinical Evidence and Therapeutic Implications. Trends Cardiovasc. Med..

[104] Scioli M.G., Storti G., D’Amico F., Rodríguez Guzmán R., Centofanti F., Doldo E., Céspedes Miranda E.M., Orlandi A. (2020). Oxidative Stress and New Pathogenetic Mechanisms in Endothelial Dysfunction: Potential Diagnostic Biomarkers and Therapeutic Targets. J. Clin. Med..

[105] Rocha E.M., De Miranda B., Sanders L.H. (2018). Alpha-Synuclein: Pathology, Mitochondrial Dysfunction and Neuroinflammation in Parkinson’s Disease. Neurobiol. Dis..

[106] Anderson J.P., Walker D.E., Goldstein J.M., de Laat R., Banducci K., Caccavello R.J., Barbour R., Huang J., Kling K., Lee M. (2006). Phosphorylation of Ser-129 Is the Dominant Pathological Modification of α-Synuclein in Familial and Sporadic Lewy Body Disease. J. Biol. Chem..

[107] Bogale T.A., Faustini G., Longhena F., Mitola S., Pizzi M., Bellucci A. (2021). Alpha-Synuclein in the Regulation of Brain Endothelial and Perivascular Cells: Gaps and Future Perspectives. Front. Immunol..

[108] Malfertheiner K., Stefanova N., Heras-Garvin A. (2021). The Concept of α-Synuclein Strains and How Different Conformations May Explain Distinct Neurodegenerative Disorders. Front. Neurol..

[109] Elabi O., Gaceb A., Carlsson R., Padel T., Soylu-Kucharz R., Cortijo I., Li W., Li J.-Y., Paul G. (2021). Human α-Synuclein Overexpression in a Mouse Model of Parkinson’s Disease Leads to Vascular Pathology, Blood Brain Barrier Leakage and Pericyte Activation. Sci. Rep..

[110] Armulik A., Genové G., Betsholtz C. (2011). Pericytes: Developmental, Physiological, and Pathological Perspectives, Problems, and Promises. Dev. Cell.

[111] Ferland-McCollough D., Slater S., Richard J., Reni C., Mangialardi G. (2017). Pericytes, an Overlooked Player in Vascular Pathobiology. Pharmacol. Ther..

[112] Araújo B., Caridade-Silva R., Soares-Guedes C., Martins-Macedo J., Gomes E.D., Monteiro S., Teixeira F.G. (2022). Neuroinflammation and Parkinson’s Disease—From Neurodegeneration to Therapeutic Opportunities. Cells.

[113] Zhang W., Xiao D., Mao Q., Xia H. (2023). Role of Neuroinflammation in Neurodegeneration Development. Signal Transduct. Target. Ther..

[114] Picca A., Calvani R., Coelho-Junior H.J., Landi F., Bernabei R., Marzetti E. (2020). Mitochondrial Dysfunction, Oxidative Stress, and Neuroinflammation: Intertwined Roads to Neurodegeneration. Antioxidants.

[115] Kowalczyk P., Sulejczak D., Kleczkowska P., Bukowska-Ośko I., Kucia M., Popiel M., Wietrak E., Kramkowski K., Wrzosek K., Kaczyńska K. (2021). Mitochondrial Oxidative Stress—A Causative Factor and Therapeutic Target in Many Diseases. Int. J. Mol. Sci..

[116] Liemburg-Apers D.C., Willems P.H.G.M., Koopman W.J.H., Grefte S. (2015). Interactions between Mitochondrial Reactive Oxygen Species and Cellular Glucose Metabolism. Arch. Toxicol..

[117] Murphy M.P. (2009). How Mitochondria Produce Reactive Oxygen Species. Biochem. J..

[118] Surmeier D.J. (2018). Determinants of Dopaminergic Neuron Loss in Parkinson’s Disease. FEBS J..

[119] Zhou Z.D., Yi L.X., Wang D.Q., Lim T.M., Tan E.K. (2023). Role of Dopamine in the Pathophysiology of Parkinson’s Disease. Transl. Neurodegener..

[120] Luo S.X., Huang E.J. (2016). Dopaminergic Neurons and Brain Reward Pathways. Am. J. Pathol..

[121] Adamu A., Li S., Gao F., Xue G. (2024). The Role of Neuroinflammation in Neurodegenerative Diseases: Current Understanding and Future Therapeutic Targets. Front. Aging Neurosci..

[122] Chen H., Li J., Huang Z., Fan X., Wang X., Chen X., Guo H., Liu H., Li S., Yu S. (2024). Dopaminergic System and Neurons: Role in Multiple Neurological Diseases. Neuropharmacology.

[123] Mishra A., Singh S., Shukla S. (2018). Physiological and Functional Basis of Dopamine Receptors and Their Role in Neurogenesis: Possible Implication for Parkinson’s Disease. J. Exp. Neurosci..

[124] Lepeta K., Lourenco M.V., Schweitzer B.C., Martino Adami P.V., Banerjee P., Catuara-Solarz S., de La Fuente Revenga M., Guillem A.M., Haidar M., Ijomone O.M. (2016). Synaptopathies: Synaptic Dysfunction in Neurological Disorders—A Review from Students to Students. J. Neurochem..

[125] Pissadaki E.K., Bolam J.P. (2013). The Energy Cost of Action Potential Propagation in Dopamine Neurons: Clues to Susceptibility in Parkinson’s Disease. Front. Comput. Neurosci..

[126] Dauer W., Przedborski S. (2003). Parkinson’s Disease. Neuron.

[127] Murali Mahadevan H., Hashemiaghdam A., Ashrafi G., Harbauer A.B. (2021). Mitochondria in Neuronal Health: From Energy Metabolism to Parkinson’s Disease. Adv. Biol..

[128] Henrich M.T., Oertel W.H., Surmeier D.J., Geibl F.F. (2023). Mitochondrial Dysfunction in Parkinson’s Disease—A Key Disease Hallmark with Therapeutic Potential. Mol. Neurodegener..

[129] Klemmensen M.M., Borrowman S.H., Pearce C., Pyles B., Chandra B. (2024). Mitochondrial Dysfunction in Neurodegenerative Disorders. Neurotherapeutics.

[130] Bergers G., Song S. (2005). The Role of Pericytes in Blood-Vessel Formation and Maintenance. Neuro. Oncol..

[131] Winkler E.A., Bell R.D., Zlokovic B.V. (2011). Central Nervous System Pericytes in Health and Disease. Nat. Neurosci..

[132] Soto-Rojas L.O., Pacheco-Herrero M., Martínez-Gómez P.A., Campa-Córdoba B.B., Apátiga-Pérez R., Villegas-Rojas M.M., Harrington C.R., de la Cruz F., Garcés-Ramírez L., Luna-Muñoz J. (2021). The Neurovascular Unit Dysfunction in Alzheimer’s Disease. Int. J. Mol. Sci..

[133] Zhang H., Wang Y., Lyu D., Li Y., Li W., Wang Q., Li Y., Qin Q., Wang X., Gong M. (2021). Cerebral Blood Flow in Mild Cognitive Impairment and Alzheimer’s Disease: A Systematic Review and Meta-Analysis. Ageing Res. Rev..

[134] Dubois B., von Arnim C.A.F., Burnie N., Bozeat S., Cummings J. (2023). Biomarkers in Alzheimer’s Disease: Role in Early and Differential Diagnosis and Recognition of Atypical Variants. Alzheimers Res. Ther..

[135] Rani S., Dhar S.B., Khajuria A., Gupta D., Jaiswal P.K., Singla N., Kaur M., Singh G., Barnwal R.P. (2023). Advanced Overview of Biomarkers and Techniques for Early Diagnosis of Alzheimer’s Disease. Cell. Mol. Neurobiol..

[136] Risacher S.L., Saykin A.J. (2021). Neuroimaging Advances in Neurologic and Neurodegenerative Diseases. Neurotherapeutics.

[137] Aramadaka S., Mannam R., Sankara Narayanan R., Bansal A., Yanamaladoddi V.R., Sarvepalli S.S., Vemula S.L. (2023). Neuroimaging in Alzheimer’s Disease for Early Diagnosis: A Comprehensive Review. Cureus.

[138] Yen C., Lin C.-L., Chiang M.-C. (2023). Exploring the Frontiers of Neuroimaging: A Review of Recent Advances in Understanding Brain Functioning and Disorders. Life.

[139] Wintermark M., Colen R., Whitlow C.T., Zaharchuk G. (2018). The Vast Potential and Bright Future of Neuroimaging. Br. J. Radiol..

[140] Yu P., Shen L., Tang L. (2025). Multimodal DTI-ALPS and Hippocampal Microstructural Signatures Unveil Stage-Specific Pathways in Alzheimer’s Disease Progression. Front. Aging Neurosci..

[141] Zhang Y.-D., Dong Z., Wang S.-H., Yu X., Yao X., Zhou Q., Hu H., Li M., Jiménez-Mesa C., Ramirez J. (2020). Advances in Multimodal Data Fusion in Neuroimaging: Overview, Challenges, and Novel Orientation. Inf. Fusion.

[142] Bischof J., Fletcher G., Verkade P., Kuntner C., Fernandez-Rodriguez J., Chaabane L., Rose L.A., Walter A., Vandenbosch M., van Zandvoort M.A.M.J. (2024). Multimodal Bioimaging across Disciplines and Scales: Challenges, Opportunities and Breaking down Barriers. npj Imaging.

[143] Gaetani L., Paolini Paoletti F., Bellomo G., Mancini A., Simoni S., Di Filippo M., Parnetti L. (2020). CSF and Blood Biomarkers in Neuroinflammatory and Neurodegenerative Diseases: Implications for Treatment. Trends Pharmacol. Sci..

[144] Chiu F.-Y., Yen Y. (2023). Imaging Biomarkers for Clinical Applications in Neuro-Oncology: Current Status and Future Perspectives. Biomark. Res..

[145] Rauf A., Badoni H., Abu-Izneid T., Olatunde A., Rahman M.M., Painuli S., Semwal P., Wilairatana P., Mubarak M.S. (2022). Neuroinflammatory Markers: Key Indicators in the Pathology of Neurodegenerative Diseases. Molecules.

[146] Kumari S., Dhapola R., Sharma P., Singh S.K., Reddy D.H. (2023). Implicative Role of Cytokines in Neuroinflammation Mediated AD and Associated Signaling Pathways: Current Progress in Molecular Signaling and Therapeutics. Ageing Res. Rev..

[147] Zhang H., He K., Zhao Y., Peng Y., Feng D., Wang J., Gao Q. (2025). FNIRS Biomarkers for Stratifying Poststroke Cognitive Impairment: Evidence From Frontal and Temporal Cortex Activation. Stroke.

[148] Kazanskiy N.L., Khonina S.N., Butt M.A. (2024). A Review on Flexible Wearables—Recent Developments in Non-Invasive Continuous Health Monitoring. Sens. Actuators A Phys..

[149] Stuart T., Hanna J., Gutruf P. (2022). Wearable Devices for Continuous Monitoring of Biosignals: Challenges and Opportunities. APL Bioeng..

[150] Habeeb M., Vengateswaran H.T., Tripathi A.K., Kumbhar S.T., You H.W., Hariyadi (2024). Enhancing Biomedical Imaging: The Role of Nanoparticle-Based Contrast Agents. Biomed. Microdevices.

[151] Chagnot A., Barnes S.R., Montagne A. (2021). Magnetic Resonance Imaging of Blood–Brain Barrier Permeability in Dementia. Neuroscience.

[152] Laviña B. (2016). Brain Vascular Imaging Techniques. Int. J. Mol. Sci..

[153] Liu A.A., Voss H.U., Dyke J.P., Heier L.A., Schiff N.D. (2011). Arterial Spin Labeling and Altered Cerebral Blood Flow Patterns in the Minimally Conscious State. Neurology.

[154] Brown G.G., Clark C., Liu T.T. (2007). Measurement of Cerebral Perfusion with Arterial Spin Labeling: Part 2. Applications. J. Int. Neuropsychol. Soc..

[155] Weinberg B.D., Kuruva M., Shim H., Mullins M.E. (2021). Clinical Applications of Magnetic Resonance Spectroscopy in Brain Tumors: From Diagnosis to Treatment. Radiol. Clin. N. Am..

[156] Hamsini B.C., Reddy B.N., Neelakantan S., Kumaran S.P. (2018). Clinical Application of MR Spectroscopy in Identifying Biochemical Composition of the Intracranial Pathologies. GABA And Glutamate—New Developments In Neurotransmission Research.

[157] Rangaprakash D., Tadayonnejad R., Deshpande G., O’Neill J., Feusner J.D. (2021). FMRI Hemodynamic Response Function (HRF) as a Novel Marker of Brain Function: Applications for Understanding Obsessive-Compulsive Disorder Pathology and Treatment Response. Brain Imaging Behav..

[158] Taylor A.J., Kim J.H., Ress D. (2018). Characterization of the Hemodynamic Response Function across the Majority of Human Cerebral Cortex. Neuroimage.

[159] Madden D.J., Bennett I.J., Burzynska A., Potter G.G., Chen N., Song A.W. (2012). Diffusion Tensor Imaging of Cerebral White Matter Integrity in Cognitive Aging. Biochim. Biophys. Acta (BBA) Mol. Basis Dis..

[160] Assaf Y., Pasternak O. (2008). Diffusion Tensor Imaging (DTI)-Based White Matter Mapping in Brain Research: A Review. J. Mol. Neurosci..

[161] Maschio C., Ni R. (2022). Amyloid and Tau Positron Emission Tomography Imaging in Alzheimer’s Disease and Other Tauopathies. Front. Aging Neurosci..

[162] Rowley P.A., Samsonov A.A., Betthauser T.J., Pirasteh A., Johnson S.C., Eisenmenger L.B. (2020). Amyloid and Tau PET Imaging of Alzheimer Disease and Other Neurodegenerative Conditions. Semin. Ultrasound CT MRI.

[163] Mattsson N., Lönneborg A., Boccardi M., Blennow K., Hansson O. (2017). Clinical Validity of Cerebrospinal Fluid Aβ42, Tau, and Phospho-Tau as Biomarkers for Alzheimer’s Disease in the Context of a Structured 5-Phase Development Framework. Neurobiol. Aging.

[164] Wu Y., Li Q., Zhang R., Dai X., Chen W., Xing D. (2021). Circulating MicroRNAs: Biomarkers of Disease. Clin. Chim. Acta.

[165] El-Daly S.M., Gouhar S.A., Abd Elmageed Z.Y. (2023). Circulating MicroRNAs as Reliable Tumor Biomarkers: Opportunities and Challenges Facing Clinical Application. J. Pharmacol. Exp. Ther..

[166] Blann A.D., Seigneur M., Steiner M., Boisseau M.R., Mccollum C.N. (1997). Circulating Endothelial Cell Markers in Peripheral Vascular Disease: Relationship to the Location and Extent of Atherosclerotic Disease. Eur. J. Clin. Investig..

[167] Zanetta L., Marcus S.G., Vasile J., Dobryansky M., Cohen H., Eng K., Shamamian P., Mignatti P. (2000). Expression of von Willebrand Factor, an Endothelial Cell Marker, Is up-Regulated by Angiogenesis Factors: A Potential Method for Objective Assessment of Tumor Angiogenesis. Int. J. Cancer.

[168] Janelidze S., Barthélemy N.R., Salvadó G., Schindler S.E., Palmqvist S., Mattsson-Carlgren N., Braunstein J.B., Ovod V., Bollinger J.G., He Y. (2024). Plasma Phosphorylated Tau 217 and Aβ42/40 to Predict Early Brain Aβ Accumulation in People Without Cognitive Impairment. JAMA Neurol..

[169] Pais M.V., Forlenza O.V., Diniz B.S. (2023). Plasma Biomarkers of Alzheimer’s Disease: A Review of Available Assays, Recent Developments, and Implications for Clinical Practice. J. Alzheimers Dis. Rep..

[170] De Caterina R., Basta G., Lazzerini G., Dell’Omo G., Petrucci R., Morale M., Carmassi F., Pedrinelli R. (1997). Soluble Vascular Cell Adhesion Molecule-1 as a Biohumoral Correlate of Atherosclerosis. Arterioscler. Thromb. Vasc. Biol..

[171] Attia E.F., Jolley S.E., Crothers K., Schnapp L.M., Liles W.C. (2016). Soluble Vascular Cell Adhesion Molecule-1 (SVCAM-1) Is Elevated in Bronchoalveolar Lavage Fluid of Patients with Acute Respiratory Distress Syndrome. PLoS ONE.

[172] Fujimoto J.G., Pitris C., Boppart S.A., Brezinski M.E. (2000). Optical Coherence Tomography: An Emerging Technology for Biomedical Imaging and Optical Biopsy. Neoplasia.

[173] Lukaszewski M., Nelke K. (2024). Near-Infrared Spectroscopy (NIRS) in the Assessment of Cerebral Tissue Oxygenation (RSO2): Methodological Issues and Dilemmas. Anesth. Res..

[174] Ali J., Cody J., Maldonado Y., Ramakrishna H. (2022). Near-Infrared Spectroscopy (NIRS) for Cerebral and Tissue Oximetry: Analysis of Evolving Applications. J. Cardiothorac. Vasc. Anesth..

[175] Pan Y., Wan W., Xiang M., Guan Y. (2022). Transcranial Doppler Ultrasonography as a Diagnostic Tool for Cerebrovascular Disorders. Front. Hum. Neurosci..

[176] Skow R.J., Brothers R.M., Claassen J.A.H.R., Day T.A., Rickards C.A., Smirl J.D., Brassard P. (2022). On the Use and Misuse of Cerebral Hemodynamics Terminology Using Transcranial Doppler Ultrasound: A Call for Standardization. Am. J. Physiol.-Heart Circ. Physiol..

[177] Mezzanotte L., van ‘t Root M., Karatas H., Goun E.A., Löwik C.W.G.M. (2017). In Vivo Molecular Bioluminescence Imaging: New Tools and Applications. Trends Biotechnol..

[178] Sato A., Klaunberg B., Tolwani R. (2004). In Vivo Bioluminescence Imaging. Comp. Med..

[179] Ahmad A., Patel V., Xiao J., Khan M.M. (2020). The Role of Neurovascular System in Neurodegenerative Diseases. Mol. Neurobiol..

[180] Kwon H.S., Koh S.-H. (2020). Neuroinflammation in Neurodegenerative Disorders: The Roles of Microglia and Astrocytes. Transl. Neurodegener..

[181] Lyman M., Lloyd D.G., Ji X., Vizcaychipi M.P., Ma D. (2014). Neuroinflammation: The Role and Consequences. Neurosci. Res..

[182] Ganguly U., Kaur U., Chakrabarti S.S., Sharma P., Agrawal B.K., Saso L., Chakrabarti S. (2021). Oxidative Stress, Neuroinflammation, and NADPH Oxidase: Implications in the Pathogenesis and Treatment of Alzheimer’s Disease. Oxid. Med. Cell. Longev..

[183] Tsai H.-Y., Chuang H.-J., Liao W.-H., Wang Y.-J., Li P.-H., Wang W.-T., Liao S.-C., Yeh C.-F., Chen P.-R., Lai T.-H. (2024). Lifestyle Modifications and Non-Pharmacological Management in Elderly Hypertension. J. Formos. Med Assoc..

[184] Ghodeshwar G.K., Dube A., Khobragade D. (2023). Impact of Lifestyle Modifications on Cardiovascular Health: A Narrative Review. Cureus.

[185] Luo H., Xiang Y., Qu X., Liu H., Liu C., Li G., Han L., Qin X. (2019). Apelin-13 Suppresses Neuroinflammation Against Cognitive Deficit in a Streptozotocin-Induced Rat Model of Alzheimer’s Disease Through Activation of BDNF-TrkB Signaling Pathway. Front. Pharmacol..

[186] Johnson M.D., Lim H.H., Netoff T.I., Connolly A.T., Johnson N., Roy A., Holt A., Lim K.O., Carey J.R., Vitek J.L. (2013). Neuromodulation for Brain Disorders: Challenges and Opportunities. IEEE Trans. Biomed. Eng..

[187] Davidson B., Bhattacharya A., Sarica C., Darmani G., Raies N., Chen R., Lozano A.M. (2024). Neuromodulation Techniques—From Non-Invasive Brain Stimulation to Deep Brain Stimulation. Neurotherapeutics.

[188] Chang B., Jiang Y., Feng C., Li B., Mei J., Niu C. (2025). Effect of Purine Diet on Prognosis of Deep Brain Stimulation for Parkinson’s Disease. Food Sci. Hum. Wellness.

[189] Singh K., Singhal S., Pahwa S., Sethi V.A., Sharma S., Singh P., Kale R.D., Ali S.W., Sagadevan S. (2024). Nanomedicine and Drug Delivery: A Comprehensive Review of Applications and Challenges. Nano-Struct. Nano-Objects.

[190] Li X., Peng X., Zoulikha M., Boafo G.F., Magar K.T., Ju Y., He W. (2024). Multifunctional Nanoparticle-Mediated Combining Therapy for Human Diseases. Signal Transduct. Target. Ther..

[191] Wang X., Wang R., Jiang L., Xu Q., Guo X. (2022). Endothelial Repair by Stem and Progenitor Cells. J. Mol. Cell. Cardiol..

[192] Yan F., Liu X., Ding H., Zhang W. (2022). Paracrine Mechanisms of Endothelial Progenitor Cells in Vascular Repair. Acta Histochem..

[193] Melo L.G., Gnecchi M., Pachori A.S., Kong D., Wang K., Liu X., Pratt R.E., Dzau V.J. (2004). Endothelium-Targeted Gene and Cell-Based Therapies for Cardiovascular Disease. Arterioscler. Thromb. Vasc. Biol..

[194] Meng Y., Wang Y., Xu J., Lu C., Tang X., Peng T., Zhang B., Tian G., Yang J. (2023). Drug Repositioning Based on Weighted Local Information Augmented Graph Neural Network. Brief. Bioinform..

